# Safety of dipeptidyl peptidase-4 inhibitors in older adults with type 2 diabetes: a systematic review and meta-analysis of randomized controlled trials

**DOI:** 10.1177/20420986211072383

**Published:** 2022-01-21

**Authors:** Katharina Doni, Stefanie Bühn, Alina Weise, Nina-Kristin Mann, Simone Hess, Andreas Sönnichsen, Dawid Pieper, Petra Thürmann, Tim Mathes

**Affiliations:** Institute for Research in Operative Medicine, School of Medicine, Faculty of Health, Witten/Herdecke University, Witten, Germany; Institute for Health Economics and Clinical Epidemiology, University of Cologne, Cologne, Germany; Institute for Research in Operative Medicine, School of Medicine, Faculty of Health, Witten/Herdecke University, Witten, Germany; Institute for Research in Operative Medicine, School of Medicine, Faculty of Health, Witten/Herdecke University, Witten, Germany; Department of Clinical Pharmacology, School of Medicine, Faculty of Health, Witten/Herdecke University, Witten, Germany; Institute for Research in Operative Medicine, School of Medicine, Faculty of Health, Witten/Herdecke University, Witten, Germany; Department of General Practice and Family Medicine, Center for Public Health, Medical University of Vienna, Vienna, Austria; Institute for Research in Operative Medicine, School of Medicine, Faculty of Health, Witten/Herdecke University, Witten, Germany; Center for Health Services Research, Brandenburg Medical School Theodor Fontane, Rüdersdorf, Germany; Faculty of Health Sciences Brandenburg, Institute for Health Services and Health System Research, Brandenburg Medical School Theodor Fontane, Rüdersdorf, Germany; Department of Clinical Pharmacology, School of Medicine, Faculty of Health, Witten/Herdecke University, Witten, Germany; Philipp Klee-Institute for Clinical Pharmacology, Helios University Hospital Wuppertal, Wuppertal, Germany; Department of Clinical Pharmacology, School of Medicine, Faculty of Health, Witten/Herdecke University, Heusnerstraße 40, 42283 Wuppertal Germany; Institute for Medical Statistics, University Medical Center Göttingen, Göttingen, Germany

**Keywords:** dipeptidyl peptidase-4 inhibitors, meta-analyses, older people, systematic review

## Abstract

**Registration:**

PROSPERO: CRD42020210645

**Introduction:**

We aimed to assess the safety of dipeptidyl peptidase-4 (DPP-4) inhibitors in older patients with type 2 diabetes with inadequate glycaemic control.

**Methods:**

We included randomized controlled trials (RCTs) in older (⩾65 years) patients with type 2 diabetes. The intervention group was randomized to treatment with any DPP-4 inhibitors. A systematic search in MEDLINE and Embase was performed in December 2020. For assessing the risk of bias, RoB 2 tool was applied. The quality of evidence was assessed using the Grading of Recommendations, Assessment, Development and Evaluation (GRADE) approach. We pooled outcomes using random effects meta-analyses.

**Results:**

We identified 16 RCTs that included 19,317 patients with a mean age of greater than 70 years. The mean HbA1c level ranged between 7.1 and 10.0 g/dl. Adding DPP-4 inhibitors to standard care alone may increase mortality slightly [risk ratio (RR) 1.04; 95% confidence interval (CI) 0.89–1.21]. Adding DPP-4 inhibitors to standard care increases the risk for hypoglycaemia (RR 1.08; 95% CI 1.01–1.16), but difference in overall adverse events is negligible. DPP-4 inhibitors added to standard care may reduce mortality compared with sulfonylureas (RR 0.88; 95% CI 0.75–1.04). DPP-4 inhibitors probably reduce the risk for hypoglycaemia compared with sulfonylureas (magnitude of effect not quantifiable because of heterogeneity) but difference in overall adverse events is negligible. There is insufficient evidence on hospitalizations, falls, fractures, renal impairment and pancreatitis.

**Conclusion:**

There is no evidence that DPP-4 inhibitors in addition to standard care decrease mortality but DPP-4 inhibitors increase hypoglycaemia risk. Second-line therapy in older patients should be considered cautiously even in drugs with a good safety profile such as DPP-4 inhibitors. In case second-line treatment is necessary, DPP-4 inhibitors appear to be preferable to sulfonylureas.

**Plain language summary:**

**Safety of dipeptidyl peptidase-4 inhibitors in older adults with type 2 diabetes**

**Introduction:**

We performed the review to assess the safety of dipeptidyl peptidase-4 (DPP-4) inhibitors in older type 2 diabetes patients with blood sugar outside the normal level.

**Methods:**

To answer the question, we searched various electronic databases. We included studies in older (⩾65 years) patients with type 2 diabetes that assessed the safety of DPP-4 inhibitors. The data from the different studies were quantitatively summarized using statistical methods. We assessed the quality of the data to judge the certainty of the findings.

**Results:**

We identified 16 studies that included 19,317 patients with a mean age greater than 70 years. The average blood sugar level of patients in the included studies was slightly or moderately increased. Adding DPP-4 inhibitors to standard care alone may increase mortality slightly. Adding DPP-4 inhibitors to standard care increases the risk for hypoglycaemia, but difference in overall adverse events is negligible. DPP-4 inhibitors added to standard care may reduce mortality compared with sulfonylureas. DPP-4s probably reduce the risk of hypoglycaemia compared with sulfonylureas (magnitude of effect not quantifiable because of heterogeneity) but difference in overall adverse events is negligible. There is insufficient evidence on hospitalizations, falls, fractures, renal impairment and pancreatitis.

**Conclusion:**

There is no evidence that DPP-4 inhibitors in addition to standard care decrease mortality but DPP-4 inhibitors increase the risk that blood sugar falls below normal. Adding DPP-4 inhibitorss to standard care in older patients should be considered cautiously even in drugs with a good safety profile such as DPP-4 inhibitors. In case additional treatment is necessary, DPP-4 inhibitors appear to be preferable to sulfonylureas.

## Introduction

In clinical practice, a large share of patients with type 2 diabetes are older adults. It can be expected that they will make up most diabetic patients in Western countries in the future.^1–3^ Older adults are at higher risk of adverse drug-related effects. They often experience more serious consequences from such reactions (e.g. hospitalization and death).^4,5^ Moreover, older adults have a higher risk to experience adverse reactions mimicking typical geriatric symptoms such as falls and delirium.^6^ Therefore, it could be questioned whether the benefits of strict glycaemic control outweigh the harms in older adults.^7^ To our knowledge, there is no high-quality evidence specifically for older adults that supports this assumption.

In clinical practice guidelines, dipeptidyl peptidase-4 (DPP-4) inhibitors are primarily recommended for second-line treatment as an alternative to other antidiabetics, especially when aiming to reduce risk for hypoglycaemia.^8,9^ Likewise, guidelines for older adults assume that DPP-4 inhibitors may reduce the risk for hypoglycaemia and therefore are considered as a preferred treatment in older patients.^10^

Previous systematic reviews showed that DPP-4 inhibitors added to standard diabetic treatment, which usually consists of metformin and lifestyle interventions, is safe and effective regarding glycaemic control.^11,12^ A systematic review of studies in older adults found that DPP-4 inhibitors may reduce hypoglycaemia but results were uncertain regarding other safety outcomes.^13^ In the view of the narrow ridge between overtreatment and undertreatment and the relevance of adverse events in older adults, reliable data on safety are of major importance to allow sufficient balancing of benefits and risks in treatment decision-making. Robust evidence is of particular interest because costs for DPP-4 inhibitors are much higher than the costs of alternative treatment options.^14^

The objective of our systematic review is two-fold. First, we aim to assess the safety of DPP-4 inhibitors as add-on therapy compared with no additional treatment in older adults with inadequate glycaemic control. Second, we aim to assess the safety of DPP-4 inhibitors compared with alternative treatments in older adults with inadequate glycaemic control.

## Methods

We registered this review in PROSPERO: CRD42020210645. There were no changes to the protocol.

This systematic review follows the reporting recommendations of the updated Preferred Reporting Items for Systematic Reviews and Meta-Analyses (PRISMA) statement.^15^

### Eligibility criteria

#### Participants

We only included studies on older patients with type 2 diabetes. We operationalized the age criterion as follows:

- Greater than or equal to 80% of the total study population aged 65 years or older.
- Subgroup analysis reports on participants aged 65 years or older.

#### Intervention

The intervention group must be treated with any type of DPP-4 inhibitors. Any dose or regimen was eligible. Trials on DPP-4 inhibitors not approved in the European Union before 2020 were excluded.

As comparator any active control, including standard care, no treatment or placebo was eligible. In studies on additional DPP-4 inhibitors treatment in combined regimens (e.g. metformin), the non-DPP-4 inhibitors treatment must be the same in all groups, so that the groups only differ regarding DPP-4 inhibitors.

#### Outcomes

We prioritized all-cause mortality, overall adverse events and hypoglycaemia as primary outcomes [critical outcomes in Grading of Recommendations Assessment, Development and Evaluation (GRADE)]. Secondary outcomes were hospitalization, discontinuation due to adverse events, falls, fractures, delirium and pancreatitis (important outcomes).

We anticipated that the effectiveness of DPP-4 inhibitors regarding glycaemic control is constant across different age subgroups and consequently would not have a shifting effect on the benefit–risk ratio.^13,16,17^ Moreover, glycaemic control is a surrogate endpoint. Although it may be important in older patients, a greater reduction in morbidity and mortality may rather result from control of other cardiovascular risk factors than from tight glycaemic control alone. Thus, a benefit for patients cannot be directly assumed when glycaemic targets are met.^10^

#### Types of studies

Only randomized controlled trials (RCTs) or subgroup analyses of RCTs on the relevant age group were eligible.

### Information sources

First, we screened the title/abstracts of the references of all systematic reviews included in a systematic review previously performed by the research group of one member of our review team.^13^

Second, we updated the electronic literature search of the previous systematic review. For this purpose, we searched MEDLINE, MEDLINE in Process, and Embase (all via Embase) for studies published from 1 December 2015 onwards. We last run the search on 11 December 2020.

In addition, we searched the reference lists of all included RCTs and all retrieved systematic reviews on the same topic.

### Search strategy

The search strategy was prepared by an experienced information specialist in collaboration with clinical experts. The full search strategy is presented in Supplement I. The searches were limited to publications in English and German. In addition, we excluded case reports, *in vitro* studies and animal experiments. The search strategy included a search filter for RCTs, a search filter for older patients and a modified generic search filter including additional specific terms for adverse events.^18–20^ The search strategy was reviewed by a second person using the PRESS checklist and validated by checking whether clearly eligible RCTs already known would have been identified.^21^

### Selection process

Two reviewers independently screened the titles and abstracts of all records identified by the literature search. Next, full-text articles of potentially relevant reports were retrieved and assessed for compliance with the eligibility criteria by two reviewers independently. Disagreements between reviewers were resolved by discussion until consensus.

Multiple reports of the same RCT were merged, so that each trial is the unit of analysis. Title/Abstract screening of the update search was performed in Rayyan.^22^

### Data collection process

Descriptive data were extracted by one reviewer and checked for accuracy by a second reviewer. Relevant outcome data were identified by two reviewers independently by marking the section in the relevant report. Subsequently, one reviewer extracted the data and a second reviewer checked the correctness. All disagreements were resolved in discussions until consensus.

### Data items

Supplement II lists all items for which we extracted data.

We extracted data on outcomes for the last available follow-up, for example, the longest observation period.

### Study risk of bias assessment

We assessed the risk of bias by using the revised Cochrane risk-of-bias tool for RCTs (RoB 2 tool).^23^ The RoB 2 tool provides a framework for assessing the risk of bias in five distinct domains on one particular outcome, that is, for each outcome separately.

In the first domain, we additionally assessed whether the subgroup consideration raised a concern regarding the randomization process (e.g. unbalanced confounders).

One of the three levels of risk of bias was assigned to each domain:

- Low risk of bias
- Some concerns
- High risk of bias

### Effect measures

All considered outcomes were dichotomous. We extracted raw data on events and number of participants for each group and calculated relative risks for all outcomes.

### Synthesis methods

#### Statistical synthesis method

We pooled data only if RCTs were sufficiently clinically and methodologically homogeneous and the *p* value of the statistical test for heterogeneity was >0.05. To describe statistical heterogeneity, we calculated prediction intervals and *I*^2^.

We pooled adverse event data separately for each comparator (placebo, no treatment, active control, standard care).

We performed an inverse variance random effects meta-analysis using the Hartung–Knapp method and the Paule-Mandel heterogeneity variance estimator.^24,25^ For outcomes for which only sparse data were available (event rate <5%, zero event studies, less than four RCTs in a meta-analysis), we additionally pooled the results using beta-binomial regression models for sensitivity analysis.^26,27^

We used the R-Package Meta for meta-analyses and SAS 9.4 for estimating the beta-binomial models.^28^ In case of heterogeneity, we synthesized results across RCTs presenting range of effects of the point estimate of the relative risk ratio.

#### Sensitivity analyses

We performed a sensitivity analysis according to risk of bias. More precisely, we excluded RCTs at high risk of bias.

### Reporting bias assessment

We planned to assess publication bias by visual inspection of funnel plots for asymmetry if at least 10 trials for each outcome would have been available.

Bias in selection of the reported results within one trial is a domain of the RoB 2 tool (see above). For RoB 2 assessment, we compared the list of outcomes reported in the protocols or methods section with the outcomes reported in the published paper.

### Certainty of evidence assessment

We rated the certainty of the body of evidence using the GRADE approach. In the GRADE assessment, evidence from RCTs starts as ‘high-certainty’ and the following criteria are applied for downgrading the certainty of evidence by one or two levels:^29^

- Risk of bias
- Imprecision
- Inconsistency
- Indirectness
- Publication bias

The rating of these criteria leads to four levels of the certainty of evidence for each of the prioritized outcomes:^30^

- High-certainty evidence: the review authors have a lot of confidence that the true effect is similar to the estimated effect.
- Moderate-certainty evidence: the review authors believe that the true effect is probably close to the estimated effect.
- Low-certainty evidence: the review authors believe that the true effect might be markedly different from the estimated effect.
- Very low-certainty evidence: the review authors believe that the true effect is probably markedly different from the estimated effect.

One reviewer judged the certainty of the evidence and a second reviewer verified the assessment. Disagreements were resolved by discussion until consensus.

The certainty of evidence and the results are presented in the ‘Summary of Findings’ (SoF) tables.^31^ The SoF tables were prepared using GRADEpro GDT.^32^ For estimating the absolute effect, we used absolute risks of the comparator group of included RCTs.

To report the findings in consideration of the certainty of evidence, we used the standardized informative statements suggested by the GRADE working group.^33^

The certainty of evidence is expressed with the following statements:

- High certainty: reduces/increases in outcome
- Moderate certainty: likely/probably reduces/increases in outcome
- Low certainty: may reduce/increase in outcome
- Very low certainty: the evidence is uncertain in outcome

## Results

### Study selection

The initial screening of publications included in the previously published systematic review^13^ identified 18 potentially relevant reports. The electronic search provided a total of 259 citations after duplicate removal. Reference screening revealed further four potentially relevant publications. Title/abstracts of these were screened and 52 potentially eligible publications remained. The screening of full-text publications resulted in 16 RCTs (21 publications) which met all eligibility criteria.^34–54^ Supplemental Figure 1 shows the study selection according to the updated PRISMA statement 2020.^15^ A list of excluded studies with primary reason for exclusion is provided in Supplement III.

### Study characteristics

Table 1 shows the study characteristics (for detailed characteristics, see Supplement IV).

**Table 1.** Characteristics of included studies.

Study	Age (years) mean IG/CG	Female *n* (%) IG/CG	HbA1c (%) mean IG/CG	BMI (kg/m^2^) mean IG/CG	Renal function IG/CG	Intervention/comparison	Outcomes
*Add-on placebo comparison*							
Barnett *et al*.^34^	74.9/74.9	46 (28.4)/30 (38.0)	7.8/7.7	29.6/29.8	eGFR (ml/min/1.73 m^2^, according to MDRD) *n* (%) Normal (⩾90) 36 (22.2)/15 (19.0) Mild impairment (60 to <90) 83 (51.2)/42 (53.2) Moderate impairment (30 to <60) 41 (25.3)/21 (26.6) Severe impairment (<30) 2 (1.2)/1 (1.3)	Intervention Linagliptin, once-daily 5 mg Comparison Placebo, once-daily 5 mg	Any AE Discontinuation* Falls Fractures Hospitalization Hypoglycaemia Mortality Pancreatitis
Bethel *et al*.^39^	78.3/78.4	288 (29.7)/360 (34.8)	7.19/7.17	29.0/28.9	eGFR (ml/min/1.73 m^2^) mean 65.3/65.7	Intervention Sitagliptin, once-daily 100 mg (or 50 mg, if the baseline eGFR was ⩾30 and <50 ml/min/1.73 m^2^) Comparison Placebo, once-daily	Fractures Hospitalization Mortality Pancreatitis
Cooper *et al*.^37^	72.2/72.0	852 (42.0)/747 (37.7)	7.9/7.8	31.0/31.0	eGFR (ml/min/1.73 m^2^) mean 49.5/49.1	Intervention Linagliptin, once-daily 5 mg, added to usual care Comparison Placebo, once-daily, 5 mg	Any AE Discontinuation Hypoglycaemia Pancreatitis Renal impairment
Kadowaki and Kondo^41^**	58.4/60.3	34 (35.4)/32 (32.7)	8.4/8.4	24.9/24.6	NR	Intervention Teneligliptin 20 mg + glimepiride Comparison Placebo	Hypoglycaemia
Ledesma *et al*.^42^	72.3/72.5	59 (39.1)/60 (39.7)	8.2/8.1	28.3/27.9	eGFR (MDRD) (ml/min/1.73 m^2^) mean 65.9/70.3	Intervention Linagliptin, once-daily, 5 mg Comparison Placebo, 5 mg	Falls Fractures Hospitalization Hypoglycaemia Mortality Pancreatitis
Leiter *et al*.^48^	71.6/71.6	1542 (35.9)/1527 (35.8)	7.5/7.4***	30.6/30.7	eGFR (ml/min/1.73 m^2^) mean 66.6/66.5	Intervention Saxagliptin, once-daily, 5 mg (normal renal function/mild impaired renal function) (eGFR >50 ml/min/1.73 m^2^) or 2.5 mg daily if they had an eGFR of ⩽50 ml/min/1.73 m^2^ Control Placebo	Any AE Discontinuation Hypoglycaemia Mortality
Schweizer and Dejager^47^	78.0/78.3	24 (48.0)/25 (45.5)	7.8/7.8	31.0/30.0	eGFR (MDRD) (ml/min/1.73 m^2^) mean 35.5/35.1	Intervention Vildagliptin, once-daily, 50 mg Comparison Placebo	Any AE Discontinuation Hypoglycaemia Mortality
Strain *et al*.^49^	75.1/74.4	66 (47.5)/86 (61.9)	7.9/7.9	29.1/30.5	GFR (MDRD) (ml/min/1.73 m^2^) *n* (%) Normal (>80): 34 (24.5)/31 (22.3) Mild (⩾50 to ⩽80): 86 (61.9)/87 (62.6) Moderate (⩾30 to <50): 19 (13.7)/21 (15.1)	Intervention Vildagliptin, twice daily (if drug-naïve and other background OAD) or once-daily (if sulphonylurea monotherapy), 50 mg Comparison Placebo, twice-daily, 50 mg	Any AE Discontinuation Hypoglycaemia Mortality Pancreatitis
*Open-label comparison*							
Chien *et al*.^36^	73.5/72.5	31 (63.3)/25 (52.1)	9.5/10.0	26.2/26.0	NR	Intervention Sitagliptin 100 mg, once-daily Comparison Only OAD	Any AE Discontinuation Hypoglycaemia
*Sulfonylurea comparison*							
Espeland *et al*.^38^	72.0/72.0	597 (40.7)/633 (42.0)	7.1/7.1	29.4/29.1	eGFR (ml/min/1.73 m^2^) mean 70.2/70.9	Intervention Linagliptin, once-daily 5 mg Comparison Glimepiride, once-daily, 1–4 mg	Any AE Discontinuation Falls Fractures Hospitalization Hypoglycaemia Mortality Pancreatitis
Hartley *et al*.^40^	70.6/70.8	104 (52.8)/114 (59.7)	7.8/7.8	29.7/29.7	NR	Intervention Sitagliptin, once-daily 100 mg (if eGFR ⩾50 ml/min/1.73 m^2^) 50 mg (if eGFR ⩾35 and <50 ml/min/1.73 m^2^) Comparison Glimepiride has started 1 mg once-daily and could be uptitrated to a maximum dose of 6 mg/day over the first 18 weeks	Any AE Discontinuation Hypoglycaemia Mortality Pancreatitis
Matthews *et al*.^43^**	57.5/57.5	733 (46.9)/718 (46.1)	7.3/7.3	31.9/31.7	Mild renal impairment**** *n* (%) 482 (30.9)/485 (31.2)	Intervention Vildagliptin, twice-daily 50 mg Comparison Glimepiride up to 6 mg/day	Hypoglycaemia
Rosenstock *et al*.^44^	70.1/69.8	120 (54.1)/123 (56.2)	7.5/7.5	29.6/30.0	eGFR (MDRD) (ml/min/1.73 m^2^) mean 73.6/72.9	Intervention Alogliptin, once-daily 25 mg Comparison Glipizide, once-daily 5 mg titrated for inadequate control to 10 mg, as needed	Any AE Discontinuation Falls Fractures Hypoglycaemia Mortality Pancreatitis
Schernthaner *et al*.^45^	72.5/72.7	143 (39.7)/132 (36.7)	7.58/7.62	BMI category (%) <25 kg/m^2^ 14.2/18.3 ⩾25 and <30 kg/m^2^ 40.8/38.1 ⩾30 kg/m^2^ 44.7/43.3	NR	Intervention Saxagliptin, once-daily 5 mg Comparison Glimepiride, once-daily 1–6 mg (uptitrated every 3 weeks in 1- or 2-mg/day increments to the optimum dose (FPG ⩽ 6.1 mmol/l), up to 6 mg/day)	Any AE Discontinuation Fractures Hypoglycaemia Mortality Pancreatitis
*Placebo comparison*							
Barzilai *et al.*^35^	71.6/72.1	54 (53)/55 (53)	7.8/7.8	30.8/ 31.1	Creatinine clearance estimated via Cockcroft–Gault (ml/min) mean 70/72	Intervention Sitagliptin, once-daily 50–100 mg (based on creatinine clearance) Comparison Placebo	Any AE Discontinuation Fractures Hypoglycaemia Mortality Renal impairment
*Metformin comparison*							
Schweizer *et al*.^46^	71.6/70.2	94 (55.6)/78 (47.0)	7.8/7.7	29.8/29.4	GFR (MDRD) (ml/min/1.73 m^2^) *n* (%) >80 (normal) 65 (38.5)/72 (43.4) ⩾50 to ⩽80 (mild impairment) 102 (60.4)/90 (54.2) ⩽30 to <50 (moderate impairment) 2 (1.2)/4 (2.4)	Intervention Vildagliptin, once-daily 100 mg Control Metformin, daily 1500 mg	Any AE Discontinuation Hypoglycaemia Mortality

AE, adverse event; BMI, body mass index; CG, control group; eGFR, estimated glomerular filtration rate; FPG, fasting plasma glucose; IG, intervention group; MDRD, Modification of Diet in Renal Disease equation; n, number; NR, not reported; OAD, oral antidiabetic agents.

* Due to AEs.

** According to the overall study population (characteristics of patients ⩾65 years were not reported separately).

*** Median.

**** GFR = 50–80 ml/min per 1.73 m^2^.

The 16 RCTs included overall 19,317 older patients. The mean age of the population included in the analysis was greater than 70 years in each RCT. In most studies, the study population comprised more men than women. The mean HbA1c at baseline ranged between 7.1 g/dl and 10.0 g/dl. In all studies, the mean body mass index (BMI) was above normal and there were participants who had reduced renal function. Background treatment for type 2 diabetes and other comedications were well balanced between groups in almost all RCTs.

Nine RCTs compared DPP-4 inhibitors as second-line treatment in patients inadequately controlled with standard care against no treatment or placebo.^34,36,37,39,41,42,47–49^ Five RCTs compared DPP-4 inhibitor second-line treatment with sulfonylureas.^38,40,43–45^ Barzilai *et al.*^35^ and Schweizer *et al.*^46^ compared DPP-4 inhibitor first-line monotherapy with placebo and metformin, respectively.

Fifteen of the 16 included studies were funded by the pharmaceutical industry. We could not find any information on funding sources for one RCT.^36^

### Risk of bias of included RCTs

The risk of bias assessment for each study is presented in Table 2, which is in picture format.

**Table 2.** Risk of bias of included studies.

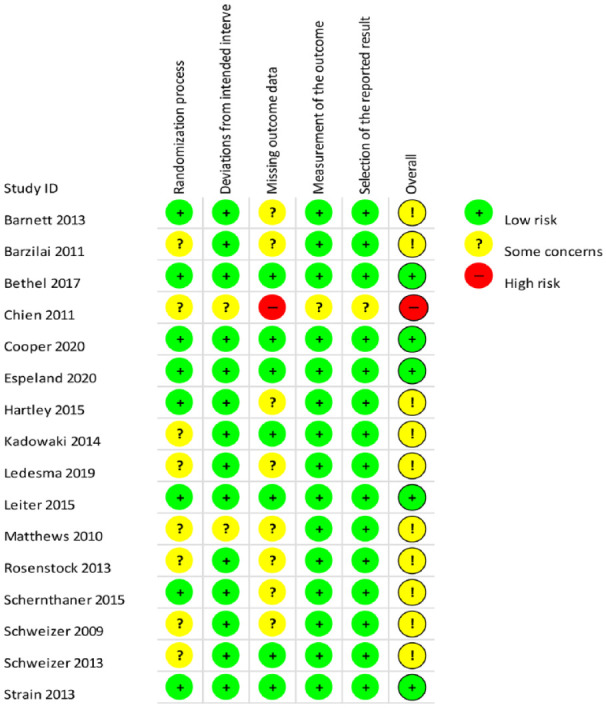

Results are presented on the study level (not on the outcome level) because in none of the included RCTs, the risk of bias varied for different outcomes (e.g. adverse events and hypoglycaemia). We rated five RCTs to be at low risk of bias.^37–39,48,49^ We had some concerns regarding risk of bias for 10 RCTs.^34,35,40–47^ One RCT we assessed as being at high risk of bias.^36^ The main reason for concerns arose in the randomization domain because allocation concealment was not clear.

Noticeably, no outcome was downrated for risk of bias in the GRADE assessment because those RCTs potentially suffering from bias had rather a smaller overall weight in meta-analyses than the RCTs at low risk of bias. Furthermore, results did not appear to be systematically different.

### Reporting bias

We could not assess reporting bias because in none of the meta-analyses, 10 RCTs or more were included.

### Effects of DPP-4 inhibitors on older patients

#### DPP-4 inhibitors as add-on to standard care alone compared with no add-on treatment

The SoF table (Table 3) shows the results of the synthesis and the certainty of evidence assessment for DPP-4 inhibitors compared with no further treatment or placebo treatment in addition to standard care alone. Results of meta-analyses and individual RCTs are presented in the forest plots (Figure 1–3 and Supplemental Figures 2–7).

**Figure 1.**
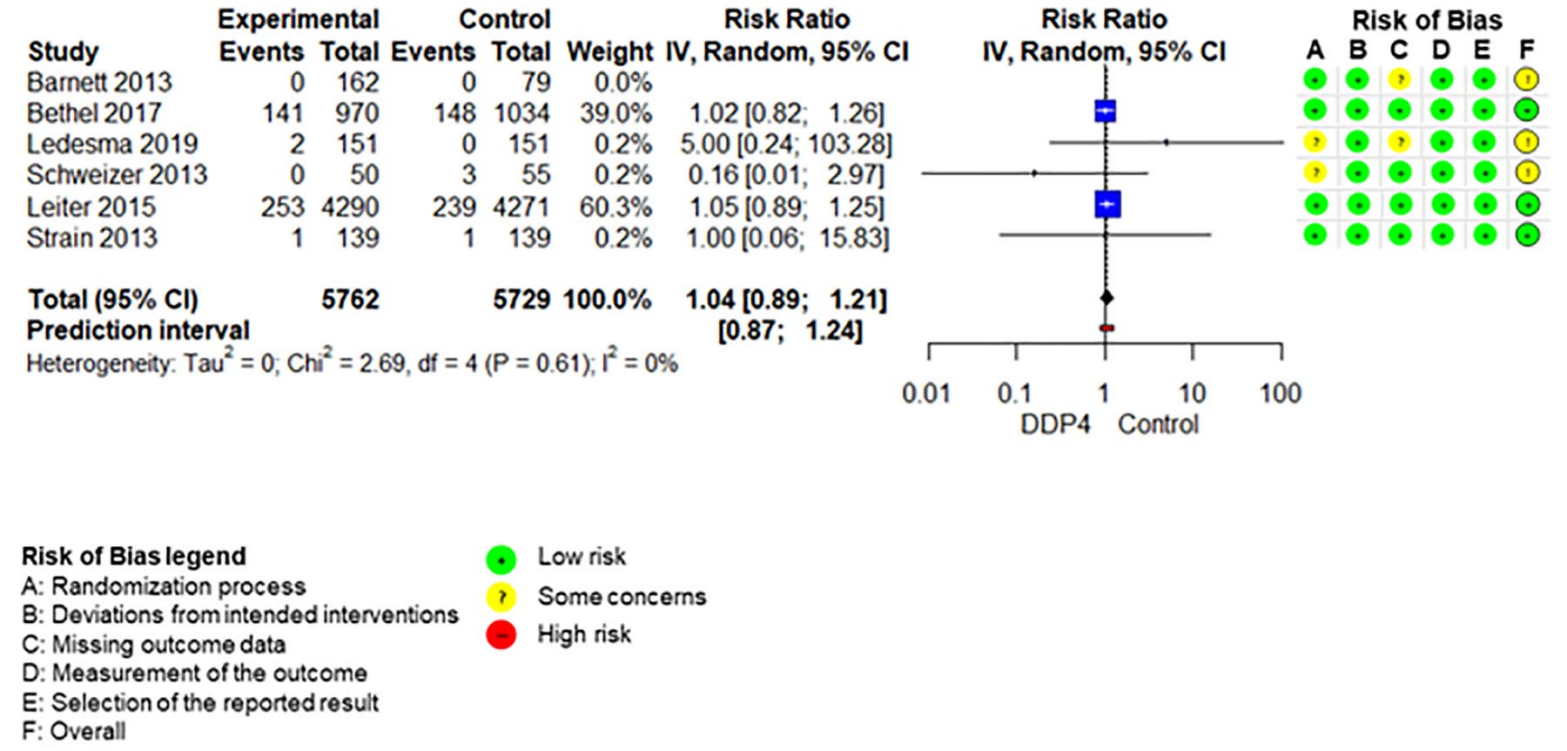
Forest plot DPP-4 inhibitors compared with no-treatment/placebo, mortality. CI, confidence interval; DPP-4, dipeptidyl peptidase-4.

**Figure 2.**
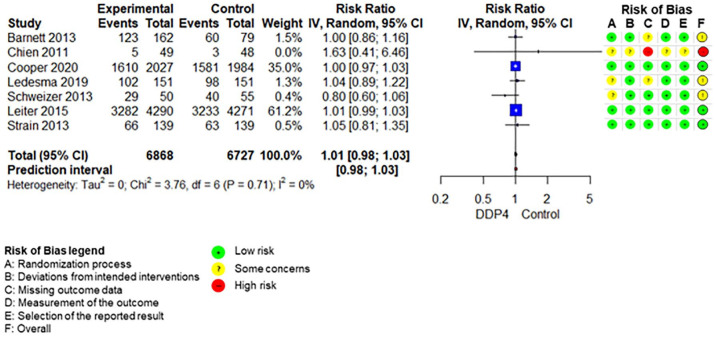
Forest plot DPP-4 inhibitors compared with no-treatment/placebo, adverse events. CI, confidence interval; DPP-4, dipeptidyl peptidase-4.

**Figure 3.**
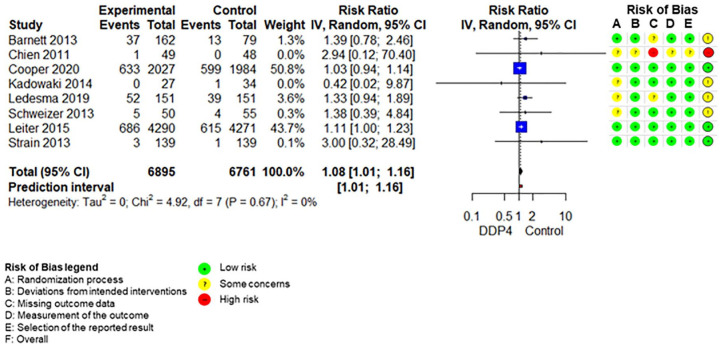
Forest plot DPP-4 inhibitors compared with no-treatment/placebo, hypoglycaemia. CI, confidence interval; DPP-4, dipeptidyl peptidase-4.

**Table 3.** GRADE summary of findings table DPP-4 inhibitors compared with no-treatment/placebo.

	Anticipated absolute effects* (95% CI)		Relative effect (95% CI)	No. of participants (studies)	Certainty of the evidence (GRADE)	Comments
	Risk with standard care	Risk with DPP-4 inhibitors as add-on				
All-cause mortality Follow-up: range 24 weeks to 5.7 years	67 per 1.000	70 per 1.000 (60–81)	RR 1.04 (0.89–1.21)	11,697 (6 RCTs)	⨁⨁◯◯ Low	Rated down for imprecision (two levels)
Any adverse events Follow-up: range 24 weeks to 2.9 years	755 per 1.000	762 per 1.000 (740–778)	RR 1.01 (0.98–1.03)	13,595 (7 RCTs)	⨁⨁⨁⨁ High	
Discontinuation due to adverse events Follow-up: range 24 weeks to 2.9 years	80 per 1.000	77 per 1.000 (67–89)	RR 0.97 (0.84–1.12)	13,294 (6 RCTs)	⨁⨁◯◯ Low	Rated down for imprecision (two levels)
Hypoglycaemia Follow-up: range 12 weeks to 2.9 years	188 per 1.000	203 per 1.000 (190–218)	RR 1.08 (1.01–1.16)	13,522 (8 RCTs)	⨁⨁⨁⨁ High	
Hospitalization Follow-up: range 24 weeks to 5.7 years	44 per 1.000	43 per 1.000 (22–84)	RR 0.99 (0.50–1.94)	2547 (3 RCTs)	⨁◯◯◯ Very low	Rated down for imprecision (two levels) and inconsistency (one level)
Falls Follow-up: 24 weeks	13 per 1.000	16 per 1.000 (0–1.000)	RR 1.25 (0.00–4320.00)	543 (2 RCTs)	⨁◯◯◯ Very low	Rated down for imprecision (two levels) and inconsistency (one level)
Fractures Follow-up: range 24 weeks to 5.7 years	36 per 1.000	41 per 1.000 (19–91)	RR 1.15 (0.52–2.53)	2566 (4 RCTs)	⨁◯◯◯ Very low	Rated down for imprecision (two levels) and inconsistency (one level)
Pancreatitis Follow-up: range 24 weeks to 5.7 years	2 per 1.000	3 per 1.000 (1–8)	RR 1.39 (0.47–4.06)	15,397 (6 RCTs)	⨁⨁◯◯ Low	Rated down for imprecision (two levels)
Renal impairment Follow-up: range 24 weeks to 2.9 years	98 per 1.000	89 per 1.000 (72–113)	RR 0.91 (0.73–1.15)	12,572 (3 RCTs)	⨁⨁◯◯ Low	Rated down for imprecision (two levels)
Delirium	No evidence for this outcome			(0 studies)	-	

* The risk in the intervention group (and its 95% CI) is based on the assumed risk in the comparison group and the relative effect of the intervention (and its 95% CI).

CI, confidence interval; DPP-4, dipeptidyl peptidase-4; GRADE, Grading of Recommendations, Assessment, Development and Evaluation; RCTs, randomized controlled trials; RR, risk ratio.

GRADE Working Group grades of evidence

High certainty: We are very confident that the true effect lies close to that of the estimate of the effect.

Moderate certainty: We are moderately confident in the effect estimate: The true effect is likely to be close to the estimate of the effect, but there is a possibility that it is substantially different.

Low certainty: Our confidence in the effect estimate is limited: The true effect may be substantially different from the estimate of the effect.

Very low certainty: We have very little confidence in the effect estimate: The true effect is likely to be substantially different from the estimate of effect.

Adding DPP-4 inhibitors to standard care may increase mortality slightly in older type 2 diabetes patients with inadequate glycaemic control.^34,39,42,47–49^ DPP-4 inhibitors increase the risk for hypoglycaemia.^34,36,37,41,42,47–49^ Differences in overall adverse events ^34,36,37,42,47–49^ and discontinuation due to adverse events^34,36,37,47–49^ are negligible. DPP-4 inhibitors may increase the risk for pancreatitis^34,36,37,47–49^ but may reduce the risk for renal impairment^37,48^ slightly. It is unclear whether DPP-4 inhibitors have an impact on hospitalization,^34,39,42^ falls,^34,42^ fractures^34,39,42^ and delirium compared with no add-on treatment because either no study assessed these predefined outcomes or the quality of evidence was very low.

Excluding the RCT at high risk of bias in the sensitivity analyses on primary outcomes does not change the results.

#### DPP-4 inhibitors as add-on to standard care compared with sulfonylureas as add-on to standard care

**Figure 4.**
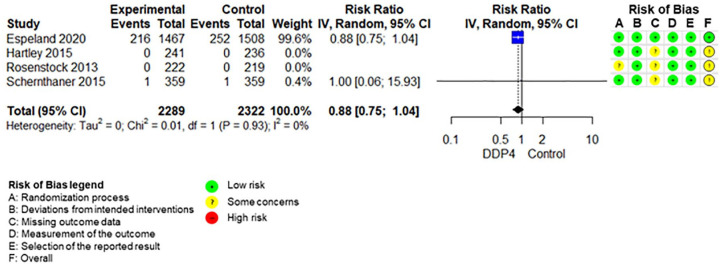
Forest plot DPP-4 inhibitors compared with sulfonylureas, mortality. CI, confidence interval; DPP-4, dipeptidyl peptidase-4.

**Figure 5.**
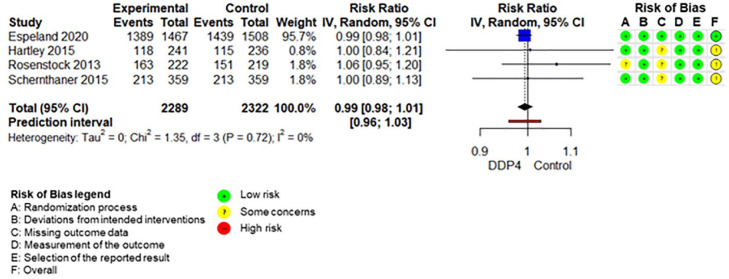
Forest plot DPP-4 inhibitors compared with sulfonylureas, adverse events. CI, confidence interval; DPP-4, dipeptidyl peptidase-4.

**Figure 6.**
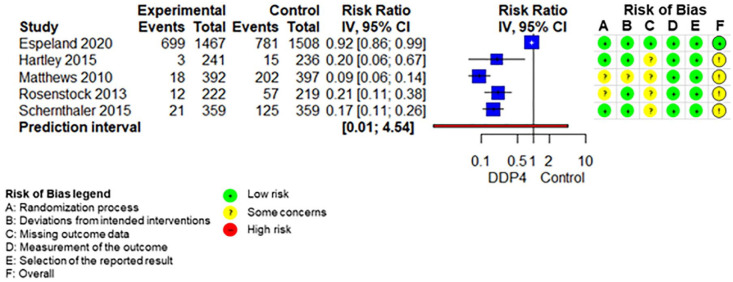
Forest plot DPP-4 inhibitors compared with sulfonylureas, hypoglycaemia. CI, confidence interval; DPP-4, dipeptidyl peptidase-4.

The SoF table (Table 4) shows the results of the synthesis and the certainty of evidence assessment for DPP-4 inhibitors added to standard care compared with sulfonylureas added to standard care. Results of the meta-analyses and individual RCTs are presented in the forest plots (Figure 4–6 and Supplemental Figures 8–11) and Supplement V. Results for RCTs which were not included in any meta-analyses because there was only one RCT reporting on the respective outcome are presented in Supplemental Table VI and VII.

**Table 4.** GRADE summary of findings table DPP-4 inhibitors as add-on to standard care compared with sulfonylureas as add-on to standard care.

	Anticipated absolute effects* (95% CI)		Relative effect (95% CI)	No. of participants (studies)	Certainty of the evidence (GRADE)	Comments
	Risk with standard care	Risk with DPP-4 inhibitors as add-on				
All-cause mortality Follow-up: range 52 weeks to 6 years	109 per 1.000	96 per 1.000 (82–113)	RR 0.88 (0.75–1.04)	4611 (4 RCTs)	⨁⨁◯◯ Low	Rated down for imprecision (two levels); results mainly based on one study and 95% CIs of the beta-binomial model were very wide.
Any adverse events Follow-up: range 24 weeks to 6 years	826 per 1.000	818 per 1.000 (809–834)	RR 0.99 (0.98–1.01)	4611 (4 RCTs)	⨁⨁⨁⨁ High	
Discontinuation due to adverse events Follow-up: range 24 weeks to 6 years	136 per 1.000	128 per 1.000 (103–158)	RR 0.94 (0.76–1.16)	5041 (4 RCTs)	⨁⨁◯◯ Low	Rated down for imprecision (one level) and inconsistency (one level)
Hypoglycaemia Follow-up: range 24 weeks to 6 years	434 per 1.000	69 per 1.000 (39–399)	RR ranged from 0.09 to 0.92	5567 (5 RCTs)	⨁⨁⨁◯ Moderate	Rated down for inconsistency (one level)
Hospitalization Follow-up: median 6 years	476 per 1.000	453 per 1.000 (415–491)	RR 0.95 (0.87–1.03)	2975 (1 RCT)	⨁⨁◯◯ Low	Rated down for imprecision (two levels)
Falls Follow-up: range 52 weeks to 6.1 years	86 per 1.000	89 per 1.000 (0–1.000)	RR 1.03 (0.00–2830.00)	3416 (2 RCTs)	⨁◯◯◯ Very low	Rated down for imprecision (two levels) and inconsistency (two levels)
Fractures Follow-up: range 52 weeks to 6 years	142 per 1.000	149 per 1.000 (41–551)	RR 1.05 (0.29–3.89)	4611 (3 RCTs)	⨁◯◯◯ Very low	Rated down for imprecision (two levels) and inconsistency (two levels)
Pancreatitis Follow-up: range 52 weeks to 6 years	4 per 1.000	4 per 1.000 (0–90)	RR 0.97 (0.05–20.85)	4611 (4 RCTs)	⨁⨁◯◯ Low	Rated down for imprecision (two levels)
Renal impairment	No evidence for this outcome (one RCT with no events)			441 (1 RCT)	-	
Delirium	No evidence for this outcome (only one study with one event)			(0 studies)	-	

* The risk in the intervention group (and its 95% CI) is based on the assumed risk in the comparison group and the relative effect of the intervention (and its 95% CI).

CI: confidence interval; DPP-4, dipeptidyl peptidase-4; GRADE, Grading of Recommendations, Assessment, Development and Evaluation; RCTs, randomized controlled trials; RR: risk ratio.

GRADE Working Group grades of evidence

High certainty: We are very confident that the true effect lies close to that of the estimate of the effect.

Moderate certainty: We are moderately confident in the effect estimate: The true effect is likely to be close to the estimate of the effect, but there is a possibility that it is substantially different.

Low certainty: Our confidence in the effect estimate is limited: The true effect may be substantially different from the estimate of the effect.

Very low certainty: We have very little confidence in the effect estimate: The true effect is likely to be substantially different from the estimate of effect.

DPP-4 inhibitors added to standard care may reduce mortality compared with sulfonylureas added to standard care in older type 2 diabetes patients with inadequate glycaemic control.^38,40,44,45^ DPP-4 inhibitors probably reduce hypoglycaemia compared with sulfonylureas, but the magnitude of the reduction cannot be reliably quantified because of heterogeneity in effect sizes between studies.^38,40,43–45^ DPP-4 inhibitors have no impact on overall adverse events^38,40,44,45^ but may reduce discontinuation due to adverse events^38,40,44,45^ slightly. DPP-4 inhibitors may reduce hospitalizations.^38^ Pancreatitis was very rare and frequencies were similar in both groups.^38,40,44,45^ It is unclear whether DPP-4 inhibitors have an impact on falls,^38,44^ fractures,^38,44,45^ renal impairment^44^ and delirium (no RCT) compared with sulfonylureas as add-on treatment to standard care because no study assessed these predefined outcomes or the quality of evidence was very low.

#### Other comparisons

The results for the RCTs that were not included in any meta-analysis because there was only one study on this comparison are shown in Supplement VII.

Barzilai *et al.*^35^ found numerically fewer overall adverse events [risk ratio (RR) 0.87; 95% confidence interval (CI) 0.66–1.15] but much more adverse events leading to discontinuation (RR 1.70; 95% CI 0.42–6.93) in the DPP-4 inhibitors group compared with the placebo group. However, statistical uncertainty was high for both outcomes. Evidence for mortality, hypoglycaemia, fractures and renal impairment was insufficient because there were no events at all or only very few events in one study arm.

Schweizer *et al.*^46^ found numerically fewer adverse events (RR 0.88; 95% CI 0.70–1.11) and much less discontinuations due to adverse events (RR 0.53; 95% CI 0.22–1.30) when taking DPP-4 inhibitors compared with metformin. Statistical uncertainty was still high for both outcomes. Evidence on hypoglycaemia was inconclusive in this RCT because only two adverse events were observed in the metformin group.

## Discussion

### Main findings and comparison with other evidence

We found that the addition of DPP-4 inhibitors in older patients with ‘inadequate’ glycaemic control may increase mortality and increases the risk of experiencing hypoglycaemia compared with standard care. Our results are consistent with a previous systematic review that included RCTs and observational studies and which showed higher cardiovascular morbidity when using DPP-4 inhibitors compared with standard care.^55^ DPP-4 inhibitors are considered to be among the preferable drugs for treating older patients with type 2 diabetes because of their low risk of hypoglycaemia.^10,56^ However, our findings point out that it may be questioned whether the cardiovascular benefits from reaching strict glycaemic targets outweigh the disadvantages arising from unintended effects in older patients even when treated with DPP-4 inhibitors. Similar concerns and the call for more individualization of antidiabetic therapy, in particular, in frail older adults have already been raised in the literature.^13,57^ However, as far as we know, no high-quality evidence exists that underpins this clinical judgement.

All RCTs comparing DPP-4 inhibitors with an active control as add-on therapy used sulfonylureas. We found that DPP-4 inhibitors may reduce the risk for mortality, hospitalization, hypoglycaemia and adverse events leading to discontinuation and may have little impact on pancreatitis suggesting that DPP-4 inhibitors have a better benefit–risk ratio than sulfonylureas in older patients. Likewise, recent systematic reviews on the safety of DPP-4 inhibitors showed that DPP-4 inhibitors are safer compared with other oral antidiabetic drugs^12^ and that DPP-4 inhibitors use may not be associated with a higher risk for pancreatitis than other antidiabetic drugs.^58^ Our analyses suggest that the main benefit of DPP-4 inhibitors arises from the avoidance of severe adverse events and reduction of risks particularly relevant for older patients (e.g. falls). The safety profile appears to be even better in older compared with younger patients,^11^ which is in particular an important finding because adverse events often have more severe consequences in older patients (e.g. falls and hospitalization because of hypoglycaemia). In agreement with expert-based guideline recommendations on treating older patients with type 2 diabetes, the generated evidence supports the judgement that safety considerations are of major importance when treating older patients.^59^ However, the high costs might be a limiting factor for prescribing DPP-4 inhibitors in many countries.^14^

### Quality of evidence and applicability of findings

The quality of the evidence was low for some outcomes. The main reason was imprecision because events were rare (e.g. pancreatitis). Moreover, we had moderate concerns regarding risk of bias in nearly half of the RCTs due to problems in the randomization domain.

Almost all studies had quite broad inclusion criteria and only few exclusion criteria (e.g. patients with end-stage renal disease) and the population appeared to be similar to real-world patients.^60,61^ Therefore, none of the studies was downrated because of concerns of applicability. Most RCTs were performed in Western countries. Therefore, the applicability of findings to other countries might be limited.

### Limitations

A limitation of this systematic review is the literature search. We decided to use the evidence from existing systematic reviews to speed up the review process. Although this could be a limitation, we anticipated that it is a reasonable shortcut considering the huge number of systematic reviews on DPP-4 inhibitors and therefore low risk of missing relevant literature when relying on previous systematic literature searches.

## Conclusion

### Implications for research

Older patients will probably make up most patients with type 2 diabetes in the near future.^1,2^ Nevertheless, there is an important research gap in the existing evidence from RCTs regarding relevant outcomes for older patients such as falls, fractures or delirium. Future studies should assess these outcomes. Otherwise, the information for judging the benefits and harms of diabetic treatment in older patients will remain incomplete due to a lack of evidence.

In addition, there is a lack of RCTs comparing DPP-4 inhibitors with other antidiabetic drugs with a presumably better safety profile than sulfonylureas (e.g. SGLT2-inhibitors) in older patients.

### Implications for practice

There is no evidence from RCTs that DPP-4 inhibitors when added to standard care decrease mortality or hospitalization in older patients. These findings indicate that initiating second-line therapy in older patients should be considered cautiously because even in drugs with a good safety profile, such as DPP-4 inhibitors, the supposed benefits of glycaemic control do not appear to outweigh the consequences from adverse events. Individualizing glycaemic targets with consideration of comorbidity, comedications and alternative measures, which could reduce the cardiovascular risk, might be a more suitable approach for treating older patients with type 2 diabetes.^10^

In case second-line treatment is necessary, DPP-4 inhibitors appear to be superior to sulfonylureas, in particular, because of the reduction of hypoglycaemia and its associated consequences, such as hospitalization.

## Supplemental Material

sj-docx-1-taw-10.1177_20420986211072383 – Supplemental material for Safety of dipeptidyl peptidase-4 inhibitors in older adults with type 2 diabetes: a systematic review and meta-analysis of randomized controlled trials Click here for additional data file. Supplemental material, sj-docx-1-taw-10.1177_20420986211072383 for Safety of dipeptidyl peptidase-4 inhibitors in older adults with type 2 diabetes: a systematic review and meta-analysis of randomized controlled trials by Katharina Doni, Stefanie Bühn, Alina Weise, Nina-Kristin Mann, Simone Hess, Andreas Sönnichsen, Dawid Pieper, Petra Thürmann and Tim Mathes in Therapeutic Advances in Drug Safety
