# Supplementary material for: Safety of dipeptidyl peptidase-4 inhibitors in older adults with type 2 diabetes: a systematic review and meta-analysis of randomized controlled trials
Source: Ther Adv Drug Saf. 2022 Jan 21;13:20420986211072383. doi: 10.1177/20420986211072383 (PMC8785305; doi:10.1177/20420986211072383)
Supplement: sj-docx-1-taw-10.1177_20420986211072383 – Supplemental material for Safety of dipeptidyl peptidase-4 inhibitors in older adults with type 2 diabetes: a systematic review and meta-analysis of randomized controlled trials [file sj-docx-1-taw-10.1177_20420986211072383.docx]

**Safety of dipeptidylpeptidase-4 inhibitors in older adults with type 2 diabetes: a systematic review and meta-analysis of randomized controlled trials**

Katharina Doni

Stefanie Bühn

Alina Weise

Nina-Kristin Mann

Simone Hess

Andreas Sönnichsen

Dawid Pieper

Petra Thürmann

Tim Mathes (corresponding author)

**Supplement I: Embase search strategy**

| ('aged'/de OR 'frail elderly'/de OR 'geriatrics'/de OR 'very elderly'/de OR 'aging'/de OR (geriatric* OR elder* OR old* OR ageing OR aging):ti,ab)  AND ('non insulin dependent diabetes mellitus'/exp OR 'non insulin dependent diabetes mellitus'/de OR (mody OR niddm OR t2dm):ti,ab OR (“non-insulin-dependent” OR “noninsulin dependent” OR “insulin independent”):ti,ab OR (“typ$ 2 diabetes” OR “typ$ ii diabetes”):ti,ab OR “ketosis resistant diabetes”:ti,ab OR ((late OR adult OR maturity) NEXT/1 “onset diabetes”):ti,ab)  NOT ('diabetes insipidus'/exp OR 'diabetes insipidus'/de OR “diabetes insipidus”:ti,ab)  AND ('dipeptidyl peptidase IV inhibitor'/exp OR 'dipeptidyl peptidase IV inhibitor'/de OR 'dipeptidyl peptidase IV'/exp OR 'dipeptidyl peptidase IV'/de OR (“dipeptidyl peptidase IV inhibitor*” OR “dipeptidyl peptidase 4 inhibitor*”):ti,ab OR gliptin*:ti,ab OR 'sitagliptin'/exp OR 'sitagliptin'/de OR 'vildagliptin'/exp OR 'vildagliptin'/de OR 'saxagliptin'/exp OR 'saxagliptin'/de OR 'alogliptin'/exp OR 'alogliptin'/de OR 'linagliptin'/exp OR 'linagliptin'/de OR (sitagliptin* OR vildagliptin* OR saxagliptin* OR alogliptin* OR linagliptin* OR janumet OR januvia OR ristaben OR tesavel OR velmetia OR xelevia OR galvus OR jalra OR xiliarx OR onglyza OR nesina OR tradjenta OR trajenta):ti,ab)  AND ((((adverse OR dangerous OR harmful OR side OR undesirable) NEXT/1 (complication* OR consequence* OR effect* OR event* OR reaction*)):ti,ab) OR 'inappropriate medication':ti,ab OR 'inappropriate medications':ti,ab OR 'inappropriate drug':ti,ab OR 'inappropriate drugs':ti,ab OR 'inappropriate prescription':ti,ab OR 'inappropriate prescribing':ti,ab OR 'potentially inappropriate medication'/de OR 'adverse drug reaction'/de OR 'quality of life'/de OR 'patient satisfaction'/de OR 'satisfaction'/de OR 'life satisfaction'/de OR 'patient preference'/de OR 'daily life activity'/de OR 'quality adjusted life year'/de OR 'personal autonomy'/de OR 'happiness'/de OR 'self-concept'/de OR 'social support'/de OR 'family relation'/de OR 'religion'/de OR 'quality of life':ti,ab OR qol:ti,ab OR 'personal autonomy':ti,ab OR 'happiness':ti,ab OR 'life quality':ti,ab OR 'quality adjusted life year*':ti,ab OR 'patient satisfaction':ti,ab OR 'patient preference*':ti,ab OR 'activities of daily living':ti,ab OR 'adl disability'/de OR 'mortality'/de OR death:ti,ab OR died:ti,ab OR mortality:ti,ab OR 'hospitalization'/de OR hospitali?ation:ti,ab OR 'functional status'/de OR headache:ti,ab OR diarrhea:ti,ab OR constipation:ti,ab OR 'cognitive impairment':ti,ab OR 'cognitive status':ti,ab OR 'functional status':ti,ab OR 'functional impairment':ti,ab OR 'life expectancy'/de OR 'drug toxicity'/de OR 'drug toxicity and intoxication'/de OR 'safety'/de OR 'patient safety'/de OR ((drug NEXT/1 (toxicity OR intoxication)):ti,ab) OR “patient safety”:ti,ab OR 'adverse event'/de OR hypoglycaemia OR 'kidney failure'/de OR (((kidney OR renal) NEXT/1 (failure OR insufficiency)):ti,ab) OR 'cardiovascular event'/exp OR “cardiovascular event”:ti,ab OR 'heart infarction'/de OR (“heart attack” OR “heart infarction” OR “myocardial infarction” OR “cardiac infarct” OR “cardiac infarction” OR “cardial infarct” OR “heart infarct” OR “myocardial infarct” OR “myocardium infarct” OR “myocardium infarction”):ti,ab OR (stroke OR apoplex* OR “cerebrovascular accident” OR cva OR “brain attack”):ti,ab OR 'falling'/de OR fall*:ti,ab OR 'delirium'/de OR (delirium OR delier OR delire OR deliria OR “deliri$us state” OR “deliri$us syndrome” OR “delirium acutum”):ti,ab)  AND (random*:ab,ti OR placebo*:de,ab,ti OR (double NEXT/1 blind*):ab,ti)  AND (english:la OR german:la)  AND ('article'/it OR 'article in press'/it OR 'review'/it)  AND ([1-12-2015]/sd NOT [31-12-2019]/sd)  NOT ('case report'/de OR 'in vitro study'/de OR 'animal experiment'/de) |
| --- |

**Supplement II: data items extracted**

1. bibliometric information:

1.1. name of first author

1.2. year of publication

2. trial-specific information:

2.1. trial name

2.2. source of funding

2.3. number and geographic regions of recruiting centers

2.4. NCT number, i.e., unique identification code given to each clinical study record registered on ClinicalTrials.gov

2.5. enrollment start and end dates

2.6. length of treatment period

2.7. length of participant follow-up

3. participant-specific information:

3.1. number of randomized participants aged 65 years or older

3.2. main inclusion and exclusion criteria

3.3. baseline characteristics of participants such as age, gender, and body-weight/Body Mass Index (BMI)

3.4. relevant clinical characteristics and comorbidities at baseline

3.5. comedication at baseline

4. intervention(s) and comparison intervention(s)

4.1. drugs with:

4.2. doses

4.3. frequency

5. outcomes:

5.1. all-cause mortality

5.4. any adverse event

5.5. discontinuation due to adverse events

5.5. Hypoglycemia

5.5. Hospitalization

5.5. Pancreatitis

5.6. renal failure

5.7. delirium

5.8. falls

5.9. length of follow-up (if necessary, for each outcome separately)

6. funding source

**Supplement III: list of excluded studies with primary reason for exclusion**

| Cahn, A., I. Raz, O. Mosenzon, G. Leibowitz, I. Yanuv, A. Rozenberg, N. Iqbal, B. Hirshberg, M. Sjostrand, C. Stahre, K. Im, E. Kanevsky, B. M. Scirica, D. L. Bhatt and E. Braunwald (2016). “Predisposing factors for any and major hypoglycemia with saxagliptin versus placebo and overall: Analysis from the savor-TIMI 53 trial.” 39(8): 1329-1337. | S |
| --- | --- |
| Cavender, M. A., B. M. Scirica, I. Raz, P. Gabriel Steg, D. K. McGuire, L. A. Leiter, B. Hirshberg, J. Davidson, A. Cahn, O. Mosenzon, K. Im, E. Braunwald and D. L. Bhatt (2016). “Cardiovascular Outcomes of Patients in SAVOR-TIMI 53 by Baseline Hemoglobin A1c.” 129(3): 340e341-340e348. | P |
| Cavender, M. A., W. B. White, Y. Liu, J. M. Massaro, R. M. Bergenstal, C. R. Mehta, F. Zannad, S. Heller, W. C. Cushman and C. P. Cannon (2018). “Total cardiovascular events analysis of the EXAMINE trial in patients with type 2 diabetes and recent acute coronary syndrome.” 41(8): 1022-1027. | P |
| Chao CT, Wang J, Wu HY, et al. Dipeptidyl peptidase 4 inhibitor use is associated with a lower risk of incident acute kidney injury in patients with diabetes. Oncotarget. 2017;8:53028–53040. | S |
| Chou, A. 2017  Effect of Long-term Incretin-Based Therapies on Ischemic Heart Diseases in Patients with Type 2 Diabetes Mellitus: A Network Meta-analysis | P |
| Cho YM, Deerochanawong C, Seekaew S, Suraamornkul S, Benjachareonwong S, Sattanon S, et al. Efficacy and safety of gemigliptin as add-on therapy to insulin, with or without metformin, in patients with type 2 diabetes mellitus (ZEUS II study). Diabetes, Obesity and Metabolism. 2020;22(1):123-7. | P |
| Del Prato S, Barnett AH, Huisman H, Neubacher D, Woerle HJ, Dugi KA. Effect of linagliptin monotherapy on glycaemic control and markers of beta-cell function in patients with inadequately controlled type 2 diabetes: a randomized controlled trial. Diabetes Obes Metab 2011; 13: 258–267. | P |
| Ferrannini E, Fonseca V, Zinman B, Matthews D, Ahren B, Byiers S, Shao Q, Dejager S. Fifty-two-week efficacy and safety of vildagliptin vs. glimepiride in patients with type 2 diabetes mellitus inadequately controlled on metformin monotherapy. Diabetes Obes Metab. 2009;11(2):157–66. | P |
| Fonseca V, Baron M, Shao Q, Dejager S. Sustained efficacy and reduced hypoglycemia during one year of treatment with vildagliptin added to insulin in patients with type 2 diabetes mellitus. Horm Metab Res. 2008;40(6):427–30. | P |
| Haak T, Meinicke T, Jones R, Weber S, von Eynatten M, Woerle HJ. Initial combination of linagliptin and metformin improves glycaemic control in type 2 diabetes: a randomized, double-blind, placebo-controlled study. Diabetes Obes Metab 2012; 14: 565–574. | P |
| Johansen OE, Neubacher D, von Eynatten M, Patel S, Woerle HJ. Cardiovascular safety with linagliptin in patients with type 2 diabetes mellitus: a pre-specified, prospective, and adjudicated meta-analysis of a phase 3 programme. Cardiovasc Diabetol. 2012;11:3. | S |
| Kaku K, Ishida K, Shimizu K, Achira M, Umeda Y. Efficacy and safety of trelagliptin in Japanese patients with type 2 diabetes with severe renal impairment or end-stage renal disease: Results from a randomized, phase 3 study. Journal of Diabetes Investigation. 2020;11(2):373-81. | P |
| McGill JB, Sloan L, Newman J et al. Long-term efficacy and safety of linagliptin in patients with type 2 diabetes and severe renal impairment: a 1-year, randomized, double-blind, placebo-controlled study. Diabetes Care 2013; 36: 237–244. | P |
| McGuire, D. K., F. Van De Werf, P. W. Armstrong, E. Standl, J. Koglin, J. B. Green, M. A. Bethel, J. H. Cornel, R. D. Lopes, S. Halvorsen, G. Ambrosio, J. B. Buse, R. G. Josse, J. M. Lachin, M. J. Pencina, J. Garg, Y. Lokhnygina, R. R. Holman and E. D. Peterson (2016). “Association between sitagliptin use and heart failure hospitalization and related outcomes in type 2 diabetes mellitus: Secondary analysis of a randomized clinical trial.” 1(2): 126-135. | P |
| McInnes, G., M. Evans, S. Del Prato, M. Stumvoll, A. Schweizer, V. Lukashevich, Q. Shao and W. Kothny (2015). “Cardiovascular and heart failure safety profile of vildagliptin: A meta-analysis of 17000 patients.” 17(11): 1085-1092. | P |
| Munch M, Meyer L, Hannedouche T, Kunz K, Alenabi F, Winiszewski P, et al. Effect of adding vildagliptin to insulin in haemodialysed patients with type 2 diabetes: The VILDDIAL study, a randomized, multicentre, prospective study. Diabetes, Obesity and Metabolism. 2020;22(6):978-87. | P |
| Owens DR, Swallow R, Dugi KA, Woerle HJ. Efficacy and safety of linagliptin in persons with type 2 diabetes inadequately controlled by a combination of metformin and sulphonylurea: a 24-week randomized study. Diabet Med 2011;28:1352e61. http: //dx.doi.org/10.1111/j.1464-5491.2011.03387.x. | P |
| Paolisso G, Monami M, Marfella R, et al. Dipeptidyl peptidase-4 inhibitors in the elderly: more benefits or risks? Adv Ther. 2012;29:218–233. | S |
| Pasquel, F. J., W. Powell, L. Peng, T. M. Johnson, S. Sadeghi-Yarandi, C. Newton, D. Smiley, M. T. Toyoshima, P. Aram and G. E. Umpierrez (2015). “A randomized controlled trial comparing treatment with oral agents and basal insulin in elderly patients with type 2 diabetes in long-term care facilities.” 3(1). | I |
| Perna, S., M. Mainardi, P. Astrone, C. Gozzer, A. Biava, R. Bacchio, D. Spadaccini, S. B. Solerte and M. Rondanelli (2018). “12-month effects of incretins versus SGLT2-inhibitors on cognitive performance and metabolic profile. A randomized clinical trial in the elderly with type-2 diabetes mellitus.” 10: 141-151. | O |
| Rosenstock, J., V. Perkovic, O. E. Johansen, M. E. Cooper, S. E. Kahn, N. Marx, J. H. Alexander, M. Pencina, R. D. Toto, C. Wanner, B. Zinman, H. J. Woerle, D. Baanstra, E. Pfarr, S. Schnaidt, T. Meinicke, J. T. George, M. Von Eynatten and D. K. McGuire (2019). “Effect of Linagliptin vs Placebo on Major Cardiovascular Events in Adults with Type 2 Diabetes and High Cardiovascular and Renal Risk: The CARMELINA Randomized Clinical Trial.” 321(1): 69-79. | P |
| Scheen AJ. Safety of dipeptidyl peptidase-4 inhibitors for treating type 2 diabetes. Expert Opin Drug Saf. 2015;14:505–524. | S |
| Schweizer, A., S. Dejager, J. E. Foley, A. Couturier, M. Ligueros-Saylan and W. Kothny (2010). “Assessing the cardio-cerebrovascular safety of vildagliptin: Meta-analysis of adjudicated events from a large Phase III type 2 diabetes population.” 12(6): 485-494. | C |
| Scirica, B. M., O. Mosenzon, D. L. Bhatt, J. A. Udell, P. G. Steg, D. K. McGuire, K. Im, E. Kanevsky, C. Stahre, M. Sjöstrand, I. Raz and E. Braunwald (2018). “Cardiovascular outcomes according to urinary albumin and kidney disease in patients with type 2 diabetes at high cardiovascular risk: Observations from the SAVOR-TIMI 53 Trial.” 3(2): 155-163. | P |
| Strózik A, Stęposz A, Basiak M, Drozdz M, Okopień B. Multifactorial effects of vildagliptin added to ongoing metformin therapy in patients with type 2 diabetes mellitus. Pharmacological Reports. 2015;67(1):24-31. | P |
| Taskinen MR, Rosenstock J, Tamminen I et al. Safety and efficacy of linagliptin  as add-on therapy to metformin in patients with type 2 diabetes: a randomized,  double-blind, placebo-controlled study. Diabetes Obes Metab 2011; 13: 65–74. | P |
| Terauchi, Y., Y. Yamada, H. Ishida, M. Ohsugi, M. Kitaoka, J. Satoh, D. Yabe, N. Shihara and Y. Seino (2017). “Efficacy and safety of sitagliptin as compared with glimepiride in Japanese patients with type 2 diabetes mellitus aged ≥ 60 years (START-J trial).” 19(8): 1188-1192. | P |
| Umpierrez, G. E., S. Cardona, D. Chachkhiani, M. Fayfman, S. Saiyed, H. Wang, P. Vellanki, J. S. Haw, D. E. Olson, F. J. Pasquel and T. M. Johnson (2018). “A Randomized Controlled Study Comparing a DPP4 Inhibitor (Linagliptin) and Basal Insulin (Glargine) in Patients With Type 2 Diabetes in Long-term Care and Skilled Nursing Facilities: Linagliptin-LTC Trial.” 19(5): 399-404.e393. | P |
| White WB, Cannon CP, Heller SR, Nissen SE, Bergenstal RM, Bakris GL, Perez AT, Fleck PR, Mehta CR, Kupfer S, et al. Alogliptin after acute coronary syndrome in patients with type 2 diabetes. N Engl J Med. 2013;369(14):1327–35. | P |
| Yki-Jarvinen H, Durán-Garcia S, Pinnetti S et al. Efficacy and safety of linagliptin  as add-on therapy to basal insulin in patients with type 2 diabetes. Diabetes  2012; 61(Suppl 1): A255. | S |

**P:** wrong patient population; **I:** wrong intervention; **S:** wrong study type **O:** no relevant outcome

**Supplement IV: data extraction sheets**

| **Study/Reference** | **Region, setting, inclusion criteria, exclusion criteria and baseline characteristics (IG/CG) of study population** | **Intervention(s), control, patient flow (IG/CG) and length of follow-up period** |
| --- | --- | --- |
| Barnett AH, Huisman H, Jones R, von Eynatten M, Patel S, Woerle HJ. Linagliptin for patients aged 70 years or older with type 2 diabetes inadequately controlled with common antidiabetes treatments: a randomised, double-blind, placebo-controlled trial. Lancet 2013; 382: 1413–1423. | **Region/Setting**  33 sites, Australia, Canada, Denmark, Netherlands, Sweden  Phase 3  March 2010 – June 2011  NCT01084005  **Inclusion criteria**   - age ≥ 70 years - type 2 diabetes mellitus - HbA1c ≥ 7.0% - stable doses of metformin, sulfonylureas, or basal insulin, or combinations of these drugs, for at least 8 weeks   **Exclusion criteria**   - FPG > 13.3 mmol/L - impaired hepatic function - previous bariatric surgery - present treatment with rapid acting or premixed insulin or systemic steroids - treatment within the previous 3 months with a thiazolidinedione, α-glucosidase inhibitor, meglitinide, GLP1 analogue, DPP4 inhibitor - myocardial infarction, stroke or TIA within 3 months prior to informed consent - treatment with anti-obesity drugs   **Patient characteristics IG/CG**  Age [y] Mean(SD)  74.9(4.4)/74.9(4.2)  Age group n(%)  <75years 91(56.2)/43(54.4)  ≥75years 71(43.8)/36(45.6)  Gender n(%)  Male 116(71.6)/49(62.0)  Female 46(28.4)/30(38.0)  Renal function eGFR [mL/min/1.73m², according to MDRD] n(%)  Normal (≥90) 36(22.2)/15(19.0)  Mild impairment (60 to <90) 83(51.2)/42(53.2)  Moderate impairment (30 to <60) 41(25.3)/21(26.6)  Severe impairment (<30) 2(1.2)/1(1.3)  HbA1c [%] Mean(SD)  7.8(0.8)/7.7(0.7)  HbA1c distribution n(%)  <7% 11(6.9)/6(7.7)  ≥7% and <8% 91(56.9)/52(66.7)  ≥8% and <9% 45(28.1)/15(19.2)  ≥9% 13(8.1)/5(6.4)  BMI [kg/m^2^] Mean(SD)  29.6(4.7)/29.8(4.5)  Co-medication n(%)  Metformin 133(83.1)/69(88.5)  Sulfonylurea 94(58.8)/43(55.1)  Insulin 35(21.9)/15(19.2)  Meglitinide 1(0.6)/0  α-glucosidase inhibitor 1(0.6)/0  Specific regimen n(%)  Metformin 43(26.9)/21(26.9)  Metformin plus sulfonylurea 64(40.0)/36(46.2)  Metformin plus sulfonylurea plus α-glucosidase inhibitor 1(0.6)/0  Metformin plus sulfonylurea plus insulin 9(5.6)/0  Metformin plus meglitinide 1(0.6)/0  Metformin plus insulin 15(9.4)/12(15.4)  Sulfonylurea 16(10.0)/6(7.7)  Sulfonylurea plus insulin 4(2.5)/1(1.3)  Insulin 7(4.4)/2(2.6)  Concomitant drugs (non-diabetes) n(%)  ≥1 159(98.1)/77(97.5)  ≥3 141(87.0)/67(84.8)  ≥5 96(59.3)/54(68.4)  Total drugs (diabetes and non-diabetes) n(%)  1 1(0.6)/0  2-3 13(8.0)/7(8.9)  4-5 38(23.5)/11(13.9)  >5 110(67.9)/61(77.2)  Co-morbidities  NR | **Intervention**  Linagliptin, once-daily  5 mg  **Control**  Placebo, once-daily  5 mg  **Randomized patients**  162/79  **Follow-up**  24 weeks |
| Barzilai N, Guo H, Mahoney EM, Caporossi S, Golm GT, Langdon RB, Williams-Herman D, Kaufman KD, Amatruda JM, Goldstein BJ, et al. Efficacy and tolerability of sitagliptin monotherapy in elderly patients with type 2 diabetes: a randomized, double-blind, placebo-controlled trial. Curr Med Res Opin. 2011;27(5):1049–58 | **Region/Setting**  52 sites, USA  Phase 3  March 2006 – March 2008  NCT00305604  **Inclusion criteria**   - age ≥ 65 years - type 2 diabetes mellitus - HbA1c 7-10% without medication, or as a result of a protocol wash-out from oral anti-hyperglycemic agents by the qualifying visit for randomization - community dwelling patients   **Exclusion criteria**   - type 1 diabetes - patients who had received insulin or exenatide within 8 weeks prior to screening - active liver disease - recent change in cardiovascular status (such as acute coronary syndrome, coronary artery intervention, worsening congestive heart failure, stroke, a transient ischemic neurologic event, or worsening symptoms of coronary artery disease) - eCrCl <35 mL/min   **Patient characteristics IG/CG**  Age [y] Mean(SD)  71.6(6.1)/72.1(6.0)  Gender n(%)  Male 48(47)/49(47)  Female 54(53)/55(53)  HbA1c [%] Mean(SD)  7.8(0.8)/7.8(0.7)  HbA1c distribution n(%)  <8.0% 69(68)/68(65)  ≥8.0% and < 9.0% 20(20)/30(29)  ≥9% 13(13)/6(6)  BMI [kg/m^2^] Mean(SD)  30.8(5.9)/ 31.1(7.2)  Creatinine clearance estimated via Cockcroft–Gault [mL/min] Mean(SD)  70(18)/72(20)  Co-medication  NR  Co-morbidities  NR | **Intervention**  Sitagliptin, once-daily  50-100 mg (based on creatinine clearance)  **Control**  Placebo  **Randomized patients**  102/104  **Follow-up**  24 weeks |
| Chien MN, Lee CC, Chen WC, Liu SC, Leung CH, Wang CH. Effect of sitagliptin as add-on therapy in elderly type 2 diabetes patients with inadequate glycemic control in Taiwan. Int J Gerontol. 2011;5(2):103–6. | **Region/Setting**  Taiwan  **Inclusion criteria**   - age ≥ 65 years - type 2 diabetes mellitus - OAD combinations therapy, but inadequate response - HbA1c ≥ 7%   **Exclusion criteria**   - heart failure - hepatic dysfunction - renal insufficiency (creatinine ≥ 1.5 mg/dL) - use of insulin pumps - patients receiving any educational program for diabetes management from elsewhere other than the program of the study   **Patient characteristics IG/CG**  Age [y] Mean(SD)  73.5(5.9)/72.5(5.2)  Gender n(%)  Male 18(36.7)/23(47.9)  Female 31(63.3)/25(52.1)  HbA1c [%] Mean(SD)  9.5(1.3)/10.0(1.6)  BMI [kg/m^2^] Mean(SD)  26.2(2.2)/26.0(2.3)  Co-medication  NR  Co-morbidities  NR | **Intervention**  OAD + Sitagliptin 100 mg, once-daily  **Control**  Only OAD  **Randomized patients**  49/48  **Follow-up**  24 weeks |
| Rosenstock J, Perkovic V, Johansen OE, Cooper ME, Kahn SE, Marx N, Alexander JH, Pencina M, Toto RD, Wanner C, Zinman B, Woerle HJ, Baanstra D, Pfarr E, Schnaidt S, Meinicke T, George JT, von Eynatten M, McGuire DK; CARMELINA Investigators. Effect of Linagliptin vs Placebo on Major Cardiovascular Events in Adults With Type 2 Diabetes and High Cardiovascular and Renal Risk: The CARMELINA Randomized Clinical Trial. JAMA. 2019 Jan 1;321(1):69-79. doi: 10.1001/jama.2018.18269. PMID: 30418475; PMCID: PMC6583576.  **Cooper, M. E., et al. (2020). "Cardiovascular and kidney outcomes of linagliptin treatment in older people with type 2 diabetes and established cardiovascular disease and/or kidney disease: A prespecified subgroup analysis of the randomized, placebo-controlled CARMELINA® trial." Diabetes, Obesity and Metabolism 22(7): 1062-1073.** | **Region/Setting**  605 clinical sites in 27 countries  August 2013 – January 2018  CARMELINA, NCT01897532  I**nclusion criteria**   - Type 2 diabetes mellitus - age ≥ 18 years at screening - glycated haemoglobin A1c (HbA1c) level between 6.5% and 10.0% inclusive - body mass index (BMI) ≤45 kg/m^2^ - established CVD (previous myocardial infarction or stroke, and/or current coronary, carotid or peripheral artery disease) together with an urinary albumin-to-creatinine ratio (UACR) >30 mg/g, - and/or impaired kidney function [estimated glomerular filtration rate (eGFR) 15 to <45 mL/min/1.73 m2 or eGFR ≥45 to 75 mL/min/1.73 m2 with UACR >200 mg/g] - drug-naïve patients or patients on glucose-lowering pharmacotherapies - (unless the latter were taking DPP4 inhibitors, glucagon-like peptide-1 (GLP-1) receptor agonists and/or sodiumglucose co-transporter-2 (SGLT2) inhibitors)   E**xclusion criteria**   - Participants with end-stage renal disease (defined as an eGFR less than 15 mL/min/1.73 m2 or requiring maintenance dialysis)   **Patient characteristics age category 65 to <75 years IG/CG**  Age group n(%)  65 to <75 years 2800(40.1)  ≥75 years 1211(17.4)  Age [y] Mean(SD)  69.2( 2.8)/ 69.1(2.9)  Gender n(%)  Male 838(59.6)/901(64.6)  Female 567(40.4)/494(35.4)  HbA1c [%] Mean(SD)  7.9(1.0)/7.9(1.0)  BMI[kg/m^2^] Mean(SD)  31.2(5.4)/31.4(5.3)  Renal function eGRF, mean(SD)  51.4(21.7)/51.1(21.7)  Renal function eGFR, n(%)  ≥ 90 mL/min/1.73m^2^ 75(5.3)/80(5.7)  ≥ 60 mL/min/1.73m^2^ 455(32.4)/462(33.1)  ≥ 45 to <60 mL/min/1.73m^2^ 299(21.3)/297(21.3)  ≥ 30 to <45 mL/min/1.73m 439(31.2)/419(30.0)  < 30 mL/min/1.73m^2^ 212(15.1)/217(15.6)  Renal function UACR, median (IQR)  139(39–617)/155(42–719)  Renal function UACR, n(%)  < 30 mg/g 299(21.3)/290(20.8)  30-300 mg/g 604(43.0)/587(42.1)  > 300 mg/g 502(35.7)/518(37.1)  Missing 0/0  **Co-medication n(%)**  Glucose-lowering medication, n(%)  Metformin 732(52.1)/749(53.7)  Sulphonylurea 430(30.6)/460(33.0)  Insulin 832(59.2)/823(59.0)  GLP-1 receptor agonist 0/0  SGLT2 inhibitor 0/0  Antihypertensive medication, n(%)  ACE inhibitor or ARB 1147(81.6)/1146(82.2)  β-blocker 839(59.7)/862(61.8)  Diuretic 780(55.5)/806(57.8)  Calcium antagonist 613(43.6)/609(43.7)  Other medication, n(%)  Aspirin 878(62.5)/891(63.9)  Statins 1001(71.2)/1035(74.2)  Co-morbidities n(%)  History of heart failure 390(27.8)/378(27.1)  Ischaemic heart disease 852(60.6)/847(60.7)  History of hypertension 1276(90.8)/1288(92.3)  Atrial fibrillation 153(10.9)/164(11.8)  **Patient characteristics age category ≥75years IG/CG**  Age group n(%)  65 to <75 years 2800(40.1)  ≥75 years 1211(17.4)  Age [y] Mean(SD)  78.9(3.4)/ 78.8(3.5)  Gender n(%)  Male 337(54.2)/336(57.0)  Female 285(45.8)/ 253(43.0)  HbA1c [%] Mean(SD)  7.8(0.9)/7.7(1.0)  BMI[kg/m^2^] Mean(SD)  30.4(4.7)/30.2(5.0)  Renal function eGRF, mean(SD)  45.3(18.1)/44.2(17.2)  Renal function eGFR, n(%)  ≥ 90 mL/min/1.73m2 17(2.7)/9(1.5)  ≥ 60 mL/min/1.73m2 119(19.1)/103(17.5)  ≥ 45 to <60 mL/min/1.73m2 138(22.2)/124(21.1)  ≥ 30 to <45 mL/min/1.73m2 257(41.3)/252(42.8)  < 30 mL/min/1.73m2 108(17.4)/110(18.7)  Renal function UACR, median (IQR)  104(27-405)/96.4(23-349)  Renal function UACR, n(%)  < 30 mg/g 164(26.4)/175(29.7)  30-300 mg/g 278(44.7)/248(42.1)  > 300 mg/g 179(28.8)/166(28.2)  Missing 1(0.2)/0  **Co-medication n(%)**  Glucose-lowering medication, n(%)  Metformin 256(41.2)/253(43.0)  Sulphonylurea 202(32.5)/184(31.2)  Insulin 355(57.1)/334(56.7)  GLP-1 receptor agonist 0/0  SGLT2 inhibitor 0/0  Antihypertensive medication, n(%)  ACE inhibitor or ARB 505(81.2)/452(76.7)  β-blocker 374(60.1)/355(60.3)  Diuretic 400(64.3)/362(61.5)  Calcium antagonist 267(42.9)/254(43.1)  Other medication, n(%)  Aspirin 371(59.6)/341(57.9)  Statins 464(74.6)/422(71.6)  Co-morbidities n(%)  History of heart failure 175(28.1)/151(25.6)  Ischaemic heart disease 354(56.9)/321(54.5)  History of hypertension 578(92.9)/542(92.0)  Atrial fibrillation 93(15.0)/109(18.5) | **Intervention**  Linagliptin, once daily  5 mg, added to usual care  **Control**  Placebo  **Age group 65 to <75y**  Randomized patients n  1405/1395  Follow-up [y] Median  2.2/2.2  **Age group (≥75 y)**  Randomized patients (≥75 y) n  622/589  Follow-up [y] Median  2.2/2.2 |
| Rosenstock J, Kahn SE, Johansen OE, Zinman B, Espeland MA, Woerle HJ, Pfarr E, Keller A, Mattheus M, Baanstra D, Meinicke T, George JT, von Eynatten M, McGuire DK, Marx N; CAROLINA Investigators. Effect of Linagliptin vs Glimepiride on Major Adverse Cardiovascular Outcomes in Patients With Type 2 Diabetes: The CAROLINA Randomized Clinical Trial. JAMA. 2019 Sep 19;322(12):1155–66. doi: 10.1001/jama.2019.13772. Epub ahead of print. Erratum in: JAMA. 2019 Dec 3;322(21):2138. PMID: 31536101; PMCID: PMC6763993.  **Espeland MA, Pratley RE, Rosenstock J, Kadowaki T, Seino Y, Zinman B, Marx N, McGuire DK, Andersen KR, Mattheus M, Keller A, Weber M, Johansen OE. Cardiovascular outcomes and safety with linagliptin, a dipeptidyl peptidase-4 inhibitor, compared with the sulphonylurea glimepiride in older people with type 2 diabetes: A subgroup analysis of the randomized CAROLINA trial. Diabetes Obes Metab. 2020 Nov 12. doi: 10.1111/dom.14254. Epub ahead of print. PMID: 33185002.** | **Region/Setting**  607 hospital and primary care sites in 43 countries  November 2010 – August 2018  CAROLINA, NCT01243424  **Inclusion criteria**   - Adults with type 2 diabetes - Age: ≥ 40 years and ≤ 80 years - Glycated hemoglobin of 6.5 % to 8.5 % - Elevated cardiovascular risk (defined as documented atherosclerotic cardiovascular disease, multiple cardiovascular risk factors, aged at least 70 years, and evidence of microvascular complications) - BMI ≤ 45kg/m² - stable anti-diabetic background for at least 8 weeks before study start   **Exclusion critiera**   - Current or past use of DPP4 inhibitors - Treatment with glucagon-like peptide-1 receptor agonists, thiazolidinedione or insulin   **Patient characteristics age category 65 to <75 years IG/CG**  Age group n(%)  65 to <75 years 2.129 (35.3)  ≥75 years 846 (14.0)  Age [y] Mean(SD)  69.7(2.9)/69.6(2.8)  Gender n(%)  Male 641(60.6)/622(58.0)  Female 416(39.4)/450(42.0)  HbA1c [%] Mean(SD)  7.12(0.55)/7.10(0.55)  BMI[kg/m^2^] Mean(SD)  29.59(4.79)/29.37(4.96)  Renal function eGRF, mean(SD)  72.3(17.4)/73.3(18.4)  Renal function eGFR, n(%)  ≥ 90 mL/min/1.73m^2^ 165(15.6)/187(17.4)  ≥ 60 < 90 mL/min/1.73m^2^ 636(60.2)/650(60.6)  ≥ 30 to <60 mL/min/1.73m^2^ 250(23.7)/(231(21.5)  ≥ 15 to <30 mL/min/1.73m^2^ 4(0.4)/2(0.2)  < 15 mL/min/1.73m^2^ 1(0.1)/0  Renal function UACR, mg/g median (25^th^-75^th^ percentile)  9.7(5.3-33.6)/9.7(5.3-33.6)  Renal function UACR, n(%)  < 30 mg/g 769(72.8)/783(73.0)  30-300 mg/g 241(22.8)/246(22.9)  > 300 mg/g 45(4.3)/38(3.5)  **Co-medication n(%)**  Glucose-lowering medication, n(%)  Metformin 860(81.4)/871(81.3)  Sulphonylurea 299(28.3)/327(30.5)  Alpha-glucosidase inhibitor 30(2.8)/40(3.7)  Glinide (meglitinide) 8(0.8)/20(1.9)  Antihypertensive medication, n(%)  ACE inhibitor 459(43.5)/464(43.4)  β-blocker 453(42.9)/445(41.6)  Diuretic 414(39.2)/417(39.0)  Angiotensin receptor blockers 368(34.8)/339(31.7)  Calcium antagonist 352(33.3)/355(33.2)  Any antihypertensive medication 945(89.5)/951(88.9)  Other medication, n(%)  Aspirin 540(51.1)/547(51.1)  Statins 675(63.9)/715(66.8)  **Co-morbidities n(%)**  Previous cardiovascular disease, n(%)  380(36.0)/384(35.8)  Heart failure, n(%)  52(4.9)/52(4.9)  Atherosclerotic CV disease, n(%)  Coronary artery disease 367(34.7)/362(33.8)  Cerebrovascular disease 140(13.2)/135(12.6)  Peripheral artery occlusive disease 72(6.8)/71(6.6)  Any atherosclerotic CV disease 465(44.0)475(44.3)  Hypertension, n(%)  963(91.1)7/944(88.1)  Microvascular disease, n(%)  Diabetic neuropathy 170(16.1)/192(17.9)  Diabetic nephropathy 130(12.3)/146(13.6)  Diabetic retinopathy 74(7.0)/91(8.5)  Any microvascular disease 293(27.7)/352(32.8)  **Patient characteristics age category >75 years IG/CG**  Age group n(%)  65 to <75 years 2.129 (35.3)  ≥75 years 846 (14.0)  Age [y] Mean(SD)  77.8(2.6)/77.8(2.6)  Gender n(%)  Male 229(55.9)/253(58.0)  Female 181(44.1)/183(42.0)  HbA1c [%] Mean(SD)  7.10(0.51)/7.13(0.56)  BMI[kg/m^2^] Mean(SD)  28.94(4.51)/28.46(4.28)  Renal function eGRF, mean(SD)  64.9(17.3)/64.9(17.0)  Renal function eGFR, n(%)  ≥ 90 mL/min/1.73m^2^ 33(8.0)/33(7.6)  ≥ 60 < 90 mL/min/1.73m^2^ 211(51.5)/235(53.9)  ≥ 30 to <60 mL/min/1.73m^2^ 161(39.3)/160(36.7)  ≥ 15 to <30 mL/min/1.73m2 5(1.2)/8(1.8)  < 15 mL/min/1.73m^2^ 0/0  Renal function UACR, mg/g median (25^th^-75^th^ percentile)  15.9 (7.1–50.4)/13.3 (6.2–45.1)  Renal function UACR, n(%)  < 30 mg/g 260(63.4)/296(67.9)  30-300 mg/g 129(31.5)/108(24.8)  > 300 mg/g 20(4.9)/30(6.9)  **Co-medication n(%)**  Glucose-lowering medication, n(%)  Metformin 305(74.4)/336(77.1)  Sulphonylurea 138(33.7)/121(27.8)  Alpha-glucosidase inhibitor 13(3.2)/14(3.2)  Glinide (meglitinide) 9(2.2)/6(1.4)  Antihypertensive medication, n(%)  ACE inhibitor 160(39.0)/177(40.6)  β-blocker 168(41.0)/165(37.8)  Diuretic 184(44.9)/192(44.0)  Angiotensin receptor blockers 126(30.7)/144(33.0)  Calcium antagonist 145(35.4)/150(34.4)  Any antihypertensive medication 364(88.8)/385(88.3)  Other medication, n(%)  Aspirin 198(48.3)/214(49.1)  Statins 237(57.8)/285(65.4)  **Co-morbidities n(%)**  Previous cardiovascular disease, n(%)  138(33.7)/159(36.5)  Heart failure, n(%)  22(5.4)/35(8.0)  Atherosclerotic CV disease, n(%)  Coronary artery disease 143(34.9)/143(32.8)  Cerebrovascular disease 59(14.4)/61(14.0)  Peripheral artery occlusive disease 30(7.3)/39(8.9)  Any atherosclerotic CV disease 184(44.9)/191(43.8)  Hypertension, n(%)  361(88.0)/385(88.3)  Microvascular disease, n(%)  Diabetic neuropathy 80(19.5)/80(18.3)  Diabetic nephropathy 63(15.4)/63(14.4)  Diabetic retinopathy 28(6.8)/32(7.3)  Any microvascular disease 134(32.7)/136(31.2) | **Intervention**  Linagliptin, once daily  5 mg  **Control**  Glimepiride, once daily  1 to 4 mg  **Age group 65 to <75y**  Randomized patients n  1057/1072  Follow-up [y] Median  6.2/6.3  **Age group (≥75 y)**  Randomized patients (≥75 y) n  410/436  Follow-up [y] Median  6.1/6.1 |
| Green JB, Bethel MA, Armstrong PW, Buse JB, Engel SS, Garg J, Josse R, Kaufman KD, Koglin J, Korn S, et al. Effect of Sitagliptin on cardiovascular outcomes in type 2 diabetes. N Engl J Med. 2015;373(3):232–42.  **Bethel MA, Engel SS, Green JB, et al. Assessing the safety of sitagliptin in older participants in the Trial Evaluating Cardiovascular Outcomes with Sitagliptin (TECOS). Diabetes Care. 2017;40:494–501** | **Region/Setting**  TECOS  673 sites, 38 countries  December 2008 – March 2015  NCT00790205  **Inclusion criteria**   - age ≤ 50 years (subgroup ≥75 years) - type 2 diabetes mellitus - HbA1c 6.5% - 8.0% on stable dose(s) of one or two oral antihyperglycemic agents (metformin, pioglitazone, or sulfonylurea) or insulin (with or without metformin) - pre-existing cardiovascular disease (defined as a history of major coronary artery disease, ischemic cerebrovascular disease, or atherosclerotic peripheral arterial disease)   **Exclusion criteria**   - DPP4 inhibitor, glucagon-like peptide-1 receptor agonist, or thiazolidinedione (other than pioglitazone) during the preceding 3 months - history of ≥ 2 episodes of severe hypoglycemia during the preceding 12 months - eGFR < 30 ml/min/1.73m^2^ of body-surface area at baseline   **Patient characteristics IG/CG**  Age [y] Mean(SD)  78.3(3.0)/78.4(3.2)  Gender n(%)  Male 682(70.3)/674(65.2)  Female 288(29.7)/360(34.8)  HbA1c [%] Mean(SD)  7.19(0.46)/7.17(0.46)  BMI [kg/m^2^] Mean(SD)  29.0(4.9)/ 28.9(4.8)  eGFR [mL/min/1.73m^2^] Mean(SD)  65.3(19.0)/65.7(19.3)  eGFR ≥90 [mL/min/1.73m^2^] n(%)  95/960(9.9)/110/1023(10.8)  eGFR 60-89 [mL/min/1.73m^2^] n(%)  474/960(49.4)/489/1023(47.8)  eGFR 30-59 [mL/min/1.73m^2^] n(%)  390/960(40.6)/424/1023(41.4)  eGFR <30 [mL/min/1.73m^2^] n(%)  1/960(<1.0)/0  **Co**-**medication**  Antidiabetic n(%)  Metformin 711(73.3)/738(71.4)  Sulfonylurea 442(45.6)/504(48.7)  Thiazolidinedione 24(2.5)/33(3.2)  Insulin 255/717(35.6)/246/728(33.8)  Median daily dose, units(IQR)  44.0, 28.0(72.0)/42.0, 28.5(64.0)  Monotherapy n(%)  514(53.0)/552(53.4)  Dual combination therapy n(%)  446(46.0)/467(45.2)  Antihypertensive n(%)  Beta blocker 585(60.3)/622(60.2)  ACE inhibitor 506(52.2)/512(49.5)  Angiotensin receptor blocker 293(30.2)/322(31.1)  Calcium channel blocker 395(40.7)/423(40.9)  Diuretic 453(46.7)/514(49.7)  Antiplatelet n(%)  Aspirin 702(72.4)/771(74.6)  Clopidogrel/Ticlopidine 195(20.1)/188(18.2)  Vitamin K antagonist 134(13.8)/121(11.7)  Any antiplatelet 771(79.5)/820(79.3)  Lipid lowering n(%)  Statin 761(78.5)/821(79.4)  Fibrate 54(5.6)/45(4.4)  Niacin 27(2.8)/23(2.2)  Ezetimibe 45(4.6)/49(4.7)  Any lipid lowering 797(82.2)/844(81.6)  **Co-morbidities**  Prior cardiovascular disease n(%)  ≥50% stenosis in a coronary artery 493(50.8)/543(52.5)  Myocardial infarction 402(41.4)/421(40.7)  Prior PCI 355/956(37.1)/388/1013(38.3)  CABG 298(30.7)/281(27.2)  Stroke 172(17.7)/204(19.7)  TIA 64(6.6)/55(5.3)  ≥50% stenosis in a carotid artery 84(8.7)/96(9.3)  Peripheral arterial disease 178(18.4)/174(16.8)  Prior congestive heart failure n(%)  190(19.6)/232(22.4)  Atrial fibrillation n(%)  138(14.2)/153(14.8)  Alcohol abuse n(%)  11(1.1)/12(1.2)  Depression n(%)  55(5.7)/80(7.7)  Chronic liver disease n(%)  11(1.1)/10(1.0) | **Intervention**  Sitagliptin, once-daily  100 mg (or 50 mg, if the baseline eGFR was ≥30 and <50ml/min/1.73m^2)^  **Control**  Placebo, once-daily  **Randomized patients**  970/1034  **Follow-up**  Median follow-up was 3.0 years (interquartile range, 2.3 to 3.8; maximum, 5.7). |
| Hartley P, Shentu Y, Betz-Schiff P, Golm GT, Sisk CM, Engel SS, Shankar RR. Efficacy and tolerability of Sitagliptin compared with Glimepiride in elderly patients with type 2 diabetes mellitus and inadequate Glycemic control: a randomized, double-blind, non-inferiority trial. Drugs Aging. 2015;32(6):469–76 | **Region/Setting**  85 sites  August 2010-October 2012  NCT01189890  **Inclusion criteria**   - age ≥ 65 ≤ 85 years - type 2 diabetes mellitus - inadequately controlled with diet and exercise alone (HbA1c ≥ 7.0% ≤ 9.0%) - community-dwelling patients   **Exclusion criteria**   - history of type 1 diabetes mellitus - previous treatment with a DPP4 inhibitor or with insulin or GLP-1 mimetics within 8 weeks prior to screening, or peroxisome proliferator-activated receptor-γ agonists within 16 weeks prior to screening - active liver disease - recent history of cardiovascular disease (acute coronary syndrome, coronary artery intervention, NYHA Class III/IV heart failure, stroke, transient ischemic neurologic event, or new/worsening symptoms of coronary heart disease) - inadequately controlled hypertension - severe peripheral vascular disease - triglycerides > 600 mg/dL - history of infection with HIV - history of malignancy or clinically important hematologic disorder - eGFR calculated using MDRD < 35 mL/min/1.73m^2^   **Patient characteristics IG/CG**  Age [y] Mean(SD)  70.6(4.8)/70.8(4.9)  Gender n(%)  Male 93(47.2)/77(40.3) Female 104(52.8)/114(59.7)  Renal function  NR  HbA1c [%] Mean(SD)  7.8(0.7)/7.8(0.7)  HbA1c [%] range  6.4-10.6/5.7-9.9  HbA1c distribution n(%)  < 8.0% 131(66.5)/125(65.4)  ≥ 8.0% 66(33.5)/66(34.6)  BMI[kg/m^2^] Mean(SD)  29.7(4.0)/29.7(5.1)  Co-medication  NR  Co-morbidities  NR | **Intervention**  Sitagliptin, once-daily  100 mg (if eGFR ≥50 mL/min/1.73m^2^)  50 mg (if eGFR ≥ 35 and <50 mL/min/1.73m^2^)  **Control**  Glimepiride was started at a dose of 1 mg once daily and could be up-titrated to a maximum dose of 6 mg/day over the first 18 weeks  **Randomized patients**  241/239  **Follow-up** 30 weeks |
| Kadowaki T, Kondo K. Efficacy and safety of teneligliptin added to glimepiride in Japanese patients with type 2 diabetes mellitus: a randomized, double-blind, placebo-controlled study with an open-label, long-term extension. Diabetes Obes Metab. 2014;16(5):418–25 | **Region/Setting**  37 sites, Japan  Phase 3  September 2009 - March 2011  NCT00974090  **Inclusion criteria**   - age 20 - 75 years - type 2 diabetes mellitus - Japanese patients - inadequate glycaemic control (HbA1c 7.3-10.3% at weeks −4 and −2) despite using a stable dose of glimepiride (1-4 mg/day) alone for ≥8 weeks and no other OAD for ≥8weeks before week −4 - maximum change in HbA1c ≤ 0.5% between weeks −4 and −2, and FPG ≤270 mg/dl (≤15.0 mmol/l) at week −4   **Exclusion criteria**   - type 1 diabetes - treatment for arrhythmia, serious diabetic complications (proliferative retinopathy, stage 4 or higher diabetic nephropathy, and serious diabetic neuropathy) - severe hepatic or renal disorders - serum creatinine levels > 2.0 mg/dl at week −4 and aspartate aminotransferase or alanine aminotransferase ≥ 2.5 times the upper limit of normal at week −4   **Patient characteristics* IG/CG**  Age [y] Mean(SD)  58.4(8.6)/60.3(7.8)  Gender n(%)  Male 62(64.6)/66(67.3)  Female 34(35.4)/32(32.7)  HbA1c [%] Mean(SD)  8.4(0.8)/8.4(0.8)  BMI[kg/m^2^] mean(SD)  24.9(3.6)/24.6(3.6)  Co-medication  NR  Co-morbidities  NR | **Intervention**  Teneligliptin  20mg   **Control**  Placebo  **Randomized patients**  27/34  **Follow-up**  12 weeks |
| Ledesma, G., G. E. Umpierrez, J. E. Morley, D. Lewis-D'Agostino, A. Keller, T. Meinicke, S. van der Walt and M. von Eynatten (2019). "Efficacy and safety of linagliptin to improve glucose control in older people with type 2 diabetes on stable insulin therapy: A randomized trial." **21**(11): 2465-2473. | **Region/Setting**  16 countries  Phase 4  February 2014 - November 2016  NCT02240680  **Inclusion criteria**   - age ≥ 60 years - inadequately controlled type 2 diabetes mellitus - HbA1c levels of 7.0–10.0% - BMI ≤45 kg/m2 - treatment with basal insulin (stratified by <40 IU/day and ≥40 IU/day) maintained at a stable (i.e., unchanged) dose for ≥4 weeks prior to randomization. Permitted basal insulin or biosimilar were either intermediate-acting formulations (insulin neutral protamine Hagedorn; insulin lispro protamine), or long-acting formulations (insulin degludec; insulin detemir; insulin glargine). The only permitted additional glucose-lowering therapies were metformin and/or alpha-glucosidase inhibitors, administered at a stable dose for 12 weeks prior to randomization   **Exclusion criteria**   - type 1 diabetes - any glucose-lowering therapies not included in the permitted list - any anti-obesity medication - depressed patients - cognitively impaired patients - acute coronary syndrome - indication of liver disease - history of cancer - history of bariatric surgery   **Patient characteristics IG/CG**  Age [y] Mean(SD)  72.3(5.1)/72.5(5.6)  Age group [y] n(%)  ≥60 to <70years 33(21.9)/33(21.9)  ≥70 to <75years 81(53.6)/65(43.0)  ≥75years 37(24.5)/53(35.1)  Gender n(%)  Male 92(60.9)/91(60.3)  Female 59(39.1)/60(39.7)  Renal function eGFR (MDRD) [mL/min/1.73 m^2^] Mean(SD)  65.9(20.1)/70.3(20.8)  HbA1c [%] Mean(SD)  8.2(0.8)/8.1(0.7)  HbA1c distribution n(%)  <7.0% 6(4.0)/3(2.0)  ≥7.0% to <7.5% 24(16.1)/18(12.2)  ≥7.5% to <8.0% 32(21.5)/39(26.5)  ≥8.0% to <9.0% 61(40.9)/74(50.3)  ≥9.0% 26(17.4)/13(8.8)  BMI [kg/m^2^] Mean(SD)  28.32(5.62)/27.86(5.74)  Co-medication  NR  **Co-morbidities**  Macrovascular disease n(%)  135(89.4)/119(78.8)  Macrovascular disease without hypertension 56(37.1)/53(35.1)  Coronary artery disease 44(29.1)/43(28.5)  Peripheral artery occlusive disease 9(6.0)/13(8.6)  Cerebrovascular disease 17(11.3)/8(5.3)  Hypertension 128(84.8)/115(76.2)  Microvascular disease n(%)  70(46.4)/84(55.6)  Diabetic retinopathy 38(25.2)/30(19.9)  Diabetic nephropathy 36(23.8)/41(27.2)  Diabetic neuropathy 32(21.2)/51(33.8)  Diabetic foot n (%)  6(4.0)/5(3.3)  Hyperlipidemia n(%)  114(75.5)/112(74.2) | **Intervention**  Linagliptin, once-daily  5 mg  **Control**  Placebo  5 mg  **Randomized patients**  151/151  **Follow-up**  24 weeks |
| Matthews DR, Dejager S, Ahren B, Fonseca V, Ferrannini E, Couturier A, Foley JE, Zinman B. Vildagliptin add-on to metformin produces similar efficacy and reduced hypoglycaemic risk compared with glimepiride, with no weight gain: results from a 2-year study. Diabetes Obes Metab. 2010;12(9):780–9. | **Region/Setting**  402 sites  **Inclusion criteria**   - - age 18-73 years   - type 2 diabetes mellitus   - HbA1c 6.5-8.5%   - metformin for ≥ 3 months; stable dose of ≥ 1500 mg daily for a minimum of ≥ 4 weeks prior to visit 1, dose unchanged throughout the study   - BMI 22-45 kg/m^2^   **Exclusion criteria**   - - type 1 diabetes or secondary forms of diabetes   - acute metabolic diabetic complications in the past 6 months   - acute infections that might affect blood glucose control in the 4 weeks prior to visit 1   - serious cardiac conditions   - clinically significant liver or renal disease   - alanine aminotransferase or aspartate aminotransferase >3 times ULN   - direct bilirubin > 1.3 times ULN   - serum creatinine levels ≥ 132 μmoll/l in men or ≥ 123 μmol/l in women   • clinically significant thyroid stimulating hormone outside of normal range at screening  • fasting triglycerides >7.9 mmol/l  **Patient characteristics IG/CG**  Age [y] Mean(SD)  57.5(9.07)/57.5(9.19)  Age group n(%)  <65years 1170(74.9)/1159(74.5)  ≥65years 392(25.1)/397(25.5)  Gender n(%)  Male 829(53.1)/838(53.9)  Female 733(46.9)/718(46.1)  HbA1c [%], Mean(SD)  7.3(0.7)/7.3(0.7)  BMI[kg/m^2^] Mean(SD)  31*.*9( 5*.*3)/31*.*7(5*.*3)  Mild renal impairment n(%)  482(30.9)/485(31.2)  Co-medication  NR  Co-morbidities  NR | **Intervention**  Vildagliptin, twice daily  50 mg  **Control**  Glimepiride  up to 6 mg/day  **Randomized patients**  378/379  **Follow-up**  2 years |
| Rosenstock J, Wilson C, Fleck P. Alogliptin versus glipizide monotherapy in elderly type 2 diabetes mellitus patients with mild hyperglycaemia: a prospective, double blind, randomized, 1-year study. Diabetes Obes Metab.2013;15(10):906–14 | **Region/Setting**  110 sites across 15 countries  NCT 00707993  **Inclusion criteria**   - age 65 - 90 years - type 2 diabetes mellitus - on diet and exercise therapy alone during the 2 months prior to screening with HbA1c level of 6.5–9.0% or on oral antidiabetic monotherapy with HbA1c of 6.5–8.0% - Patients were able and willing to self-monitor blood glucose with a home glucose monitor   **Exclusion criteria**  NR  **Patient characteristics IG/CG**  Age [y] Mean(SD)  70.1(4.42)/69.8(4.07)  Age group n(%)  <75years 186(83.8)/193(88.1)  ≥75years 36(16.2)/26(11.9)  Gender n(%)  Male 102(45.9)/96(43.8)  Female 120(54.1)/123(56.2)  HbA1c [%] Mean(SD) n(%)  7.50(0.703)/7.45(0.634)  HbA1c [%] Median(range)  7.50(6.3-10.4)/7.40(6.2-9.5)  BMI[kg/m^2^] Mean(SD)  29.58(4.348)/30.02(4.459)  **GFR [ml/min/1.73m^2^]**  MDRD Mean(SD)  73.62(14.762)/72.89(15.524)  Cockcroft-Gault Mean(SD)  78.30(18.090)/78.12(19.910)  Co-medication  NR  Co-morbidities  NR | **Intervention**  Alogliptin, once-daily  25 mg  **Control**  Glipizide, once-daily  5 mg titrated for inadequate control to 10mg, as needed  **Randomized patients**  222/219  **Follow-up**  52 weeks |
| Schernthaner G, Duran-Garcia S, Hanefeld M, Langslet G, Niskanen L, Ostgren CJ, Malvolti E, Hardy E. Efficacy and tolerability of saxagliptin compared with glimepiride in elderly patients with type 2 diabetes: a randomized, controlled study (GENERATION). Diabetes Obes Metab. 2015; 17(7):630–8. | **Region/Setting**  GENERATION  152 sites in 12 European countries and Mexico  Phase 3b/4  October 2009 - June 2012  NCT01006603  **Inclusion criteria**   - age ≥ 65 years - type 2 diabetes mellitus - stable metformin monotherapy (any dose) for at least 8 weeks before enrolment - HbA1c7.0% -9.0%   **Exclusion criteria**   - Type 1 diabetes - treatment with any antihyperglycaemic therapy other than metformin monotherapy < 8weeks before enrolment - treatment with systemic glucocorticoids (except for replacement therapy) or cytochrome P450 3A4 inducers - history of ketoacidosis or hyperosmolar non-ketonic coma - history of haemoglobinopathies - renal impairment (creatinine clearance<60 ml/min) - cognitive function problems - alcohol or illegal drug abuse for≤12months before enrolment - history of hypersensitivity or contraindication to the study drugs - aspartate aminotransferase levels >3 times ULN and/or alanine aminotransferase levels >3 times ULN and/or total bilirubin >34 μmol/l; and creatine kinase >10 times ULN   **Patient characteristics IG/CG**  Age [y] Mean(SD)  72.5(5.7)/72.7(5.4)  Gender n(%)  Male 217(60.3)/228(63.3)  Female 143(39.7)/132(36.7)  Renal function  NR  HbA1c [%] Mean(SD)  7.58(0.67)/7.62(0.65)  HbA1c [mmol/mol] Mean  59/60  BMI category n(%)  *<*25 kg/m^2^ 51(14.2)/66(18.3)  ≥25 and*<*30 kg/m^2^ 147(40.8)/137(38.1)  ≥30 kg/m^2^ 161(44.7)/156(43.3)  Co-medication [mg] Mean(SD)  Metformin 1,647(705)/1,572(671)  Co-morbidities n(%)  Musculoskeletal and connective tissue disorders 120(33.3)/121(33.6)  Gastrointestinal disorders 85(23.6)/82(22.8)  Reproductive system and breast disorders 52(14.4)/60(16.7)  Neoplasms 53(14.7)/49(13.6)  Hypertension 276(76.7)/279(77.5)  Coronary artery disease 31(8.6)/36(10.0)  Previous myocardial infarction 34(9.4)/20(5.6)  Cardiovascular accident 19(5.3)/21(5.8)  Stable angina 17(4.7)/21(5.8)  Lipid disorder 220(61.1)/213(59.2) | **Intervention**  Saxagliptin, once-daily  5 mg  **Control**  Glimepiride, once-daily  1 - 6 mg (uptitrated every 3 weeks in 1- or 2-mg/day increments to the optimum dose (FPG≤6.1mmol/l), up to 6mg/day)  **Randomized patients**  360/360  **Follow-up**  52 weeks |
| Schweizer A, Dejager S, Bosi E. Comparison of vildagliptin and metformin monotherapy in elderly patients with type 2 diabetes: a 24-week, double-blind, randomized trial. Diabetes Obes Metab. 2009;11(8):804–12. | **Region/Setting**  113 centres in 14 countries in Europe, the Americas and Asia  September 2006 - May 2008  NCT 2006-002466-19  **Inclusion criteria**   - age ≥ 65 years - type 2 diabetes mellitus - HbA1c 7 - 9% at screening (visit 1) - male and female patients with FPG <15 mmol/l - BMI 22–40 kg/m^2^ - no oral glucose–lowering agents for at least 12 weeks prior to screening and no oral glucose-lowering agents for more than three consecutive months at any time in the past   **Exclusion criteria**   - history of type 1 or secondary forms of diabetes - acute metabolic diabetic complications within the past 6 months - congestive heart failure requiring pharmacological treatment or myocardial infarction - unstable angina or stroke or coronary artery bypass surgery within the past 6 months - liver disease such as cirrhosis or chronic active hepatitis - renal disease or renal dysfunction suggested by elevated serum creatinine levels, in accordance with country-specific prescribing guidelines for metformin   **Patient characteristics IG/CG**  Age [y] Mean(SD)  71.6(5.2)/70.2(5.1)  Gender n(%)  Male 75(44.4)/88(53.0)  Female 94(55.6)/78(47.0)  HbA1c [%] Mean(SD)  7.8(0.6)/7.7(0.6)  HbA1c group n(%)  ≤8.0% 114(67.5)/121(72.9)  >8.0% 55(32.5)/45(27.1)  BMI [kg/m^2^] Mean(SD)  29.8(4.4)/29.4(4.6)  GFR (MDRD) [ml/min/1.73 m^2^] n(%)  >80 (normal renal function) 65(38.5)/72(43.4)  ≥50 to ≤80 (mild renal insufficiency) 102(60.4)/90(54.2)  ≤30 to <50 (moderate renal insufficiency) 2(1.2)/4(2.4)  Co-medication  NR  Co-morbidities  NR | **Intervention**  Vildagliptin, once-daily  100 mg  **Control**  Metformin, daily  1500 mg  **Randomized patients**  169/166  **Follow-up**  24 weeks |
| Lukashevich V, Schweizer A, Shao Q, Groop PH, Kothny W. Safety and efficacy of vildagliptin versus placebo in patients with type 2 diabetes and moderate or severe renal impairment: a prospective 24-week randomized placebo-controlled trial Diabetes Obes Metab. 2011;13:947–54.  **Schweizer A, Dejager S. Experience with vildagliptin in patients ≥75years with type 2 diabetes and moderate or severe renal impairment. Diabetes Ther. 2013; 4(2):257–67.** | **Region/Setting** NR  **Inclusion criteria**   - age 18–85 years (subgroup ≥75 years) - type 2 diabetes mellitus either untreated (no therapy in previous 8 weeks) or treated with a sulfonylurea, alpha-glucosidase inhibitor, thiazolidinedione, insulin, meglitinide or a combination of agents and a dose stability for ≥4 weeks before first study visit - HbA1C: 6.5 - 10% - BMI 18 - 42 kg/m^2^ - moderate or severe RI [eGFR ≥30 to <50 ml/min/1.73m^2^ and <30 ml/min/1.73 m^2^, respectively]   **Exclusion criteria**   - FPG ≥ 15 mmol/l - renal transplant - significant cardiovascular history within 6 months - active liver disease or abnormal liver tests (ALT, AST or bilirubin 2× ULN)   **Patient characteristics IG/CG**  Age [y] Mean (SD)  78.0(2.5)/78.3(2.7)  Gender n(%)  Male 26(52.0)/30(54.5)  Female 24(48.0)/25(45.5)  HbA1C [%] Mean(SD)  7.8(1.0)/7.8(0.8)  BMI [kg/m^2^] Mean(SD)  31.0(4.5) 30.0(4.4)  FPG [mmol/L] Mean(SD)  8.8(3.2)/8.2(3.1)  eGFR (MDRD) [ml/min/1.73m^2^] Mean(SD)  35.5(9.4)/35.1(9.5)  eGFR group n(%)  Moderate (≥30 to < 50) 36(72.0)/35(63.6)  Severe (<30) 14(28.0)/20(36.4)  Previous antidiabetic therapy n(%)  Drug-naïve 4(8.0)/1(1.8)  Insulin monotherapy 26(52.0)/32(58.2)  Insulin & OAD 3(6.0)/11(20.0)  OAD monotherapy 17(34.0)/9(16.4)  OAD combination therapy 0(0.0)/2(3.6)  Co-medication  ≥5 medications 42(84.0)/ 47(85.5)  Anti-hypertensive 47(94.0)/ 53(96.4) Lipid-lowering 34(68.0)/ 34(61.8)  Co-morbidities  Hypertension 48(96.0)/53(96.4)  Dyslipidemia 36(72.0)/36(65.5)  High CV risk status 26(52.0)/30(54.5) | **Intervention**  Vildagliptin, once-daily  50 mg  **Control**  Placebo  **Randomized patients**  50/55  **Follow-up**  24 weeks |
| Scirica BM, Bhatt DL, Braunwald E, Steg PG, Davidson J, Hirshberg B, Ohman P, Frederich R, Wiviott SD, Hoffman EB, et al. Saxagliptin and cardiovascular outcomes in patients with type 2 diabetes mellitus. N Engl JMed. 2013;369(14):1317–26  **Leiter LA, Teoh H, Braunwald E, Mosenzon O, Cahn A, Kumar KM, Smahelova A, Hirshberg B, Stahre C, Frederich R, et al. Efficacy and safety of saxagliptin in older participants in the SAVOR-TIMI 53 trial. Diabetes Care. 2015;38(6):1145–53** | **Region/Setting**  SAVOR- TIMI 53  788 sites, 26 countries  May 2010 - December 2011  Phase 4  NCT01107886  **Inclusion criteria**   - type 2 diabetes mellitus - HbA1c ≥6.5% ≤12.0% (based on the last measured and documented laboratory measurement within 6 months) - history of either established CV disease (patients had to be at least 40 years old and have a history of a clinical event associated with atherosclerosis involving the coronary, cerebrovascular or peripheral vascular system.) or multiple risk factors for vascular disease (patients had to be at least 55 years of age (men) or 60 years of age (women) with at least one of the following additional risk factors: dyslipidemia, hypertension, or active smoking **Exclusion criteria** - current or previous (within 6 months) incretin-based therapy - long-term dialysis - renal transplant - serum creatinine level >6.0 mg/dL   **Patient characteristics IG/CG**  Age [y] Mean(SD)  71.6(5.1)/71.6(5.2)  Gender n(%)  Male 2748(64.1)/2744 (64.2)  Female 1542(35.9)/1527(35.8)  eGFR [mL/min/1.73 m^2^] Mean(SD)  66.6(21.0)/66.5(21.1)  HbA1c [%] Median(Q1-Q3)  7.5(6.9-8.4)/7.4(6.9-8.3)  HbA1c [mmol/mol] Median(Q1-Q3)  58.5(51.9-68.3)/57.4(51.9-67.2)  BMI[kg/m^2^] Mean(SD)  Men 30.3 (5.0)/30.3 (4.9)  Women 31.4 (5.8)/31.1 (5.6)  Co-medication n(%)  Insulin 1742(40.6)/1701(39.8)  Metformin 2800(65.3)/2768(64.8)  Metformin monotherapy 818(19.1)/879(20.6)  Statin 3407(79.4)/3366(78.8)  ACEi or ARB 3375(78.7)/3427(80.2)  Co-morbidities  NR | **Intervention**  Saxagliptin, once-daily  5 mg (normal renal function / mild impaired renal function (eGFR >50 mL/ min/1.73m2) or 2.5 mg daily if they had an eGFR of ≤ 50 mL/min/1.73m^2^  **Control**  Placebo **Randomized patients**  4290/4271  **Follow-up**  The median follow-up period was 2.1 years (interquartile range, 1.8 to 2.3), and the maximum follow-up time was 2.9 years |
| Strain WD, Lukashevich V, Kothny W, Hoellinger MJ, Paldanius PM. Individualised treatment targets for elderly patients with type 2 diabetes using vildagliptin add-on or lone therapy (INTERVAL): a 24 week, randomised, double-blind, placebo-controlled study. Lancet. 2013;382(9890): 409–16 | **Region/Setting**  INTERVAL  45 outpatient centres in seven European countries (Belgium, Bulgaria, Germany, Finland, Slovakia, Spain, UK)  Phase 3  December 2010 – March 2012  NCT01257451  **Inclusion criteria**   - age ≥ 70 years - type 2 diabetes mellitus - Drug-naïve or inadequate controlled HbA1c ≥ 7% ≤10.0 - FPG < 15 mmol/L (270 mg/dL) - BMI 19 - 45kg/m^2^   **Exclusion criteria**   - insulin treatment (>7 consecutive days) - incretin-based therapies in the preceding 12 weeks - use of corticosteroids within 8 weeks - use of growth hormone within 6 months of the screening visit - acute metabolic diabetic disorders - myocardial infarction - coronary artery bypass surgery - stroke within 6 months - unstable angina within 3 months - congestive heart failure (New York Heart Association classification of III or IV) - malignancy within 5 years - liver disease - Substantial laboratory abnormalities including liver function tests, renal dysfunction as suggested by reduced glomerular filtration rate (<30 mL/min per 1·73 m²), or positive hepatitis B or C tests   **Patient characteristics IG/CG**  Age [y] Mean(SD)  75.1(4.3)/74.4(4.0)  Gender n(%)  Male 73(52.5)/53(38.1)  Female 66(47.5)/86(61.9)  HbA1c [%] Mean(SD)  7.9(0.8)/7.9(0.7)  BMI[kg/m^2^] Mean(SD)  29.1(3.8)/30.5 (4.8)  GFR (MDRD) [mL/min/1.73m^2^] n(%)  Normal (>80) 34(24.5)/31(22.3)  Mild (≥50 to ≤80) 86(61.9)/87(62.6)  Moderate (≥30 to <50) 19(13.7)/21(15.1)  Co-medication  NR  Co-morbidities  NR | **Intervention**  Vildagliptin, twice daily (if drug-naïve and other background OAD) or once-daily (if sulphonylurea monotherapy)  50 mg  **Control**  Placebo, twice daily  50mg  **Randomized patients**  139/139  **Follow-up**  24 weeks |

Abbreviations: ACE: angiotensin-converting enzyme; ARB: angiotensin-converting enzyme; BMI: body mass index; CABG: coronary artery bypass graft; CG: Control Group; DPP4: Dipeptidyl peptidase-4; eCrCl: estimated creatinine clearance; eGFR: estimated glomerular filtration rate; FPG: fasting plasma glucose; IG: Intervention Group; IQR: interquartile range; MDRD: Modification of Diet in Renal Disease equation; n: number; NR: not reported; NYHA: New York Heart Association; OAD: Oral Antidiabetic Agents; PCI: percutaneous coronary intervention; SD: Standard Deviation; TECOS: Trial Evaluating Cardiovascular Outcomes with Sitagliptin; TIA: transient ischaemic attack; ULN: upper limit of normal; y: years

*According to the overall study population (patient characteristics for the subgroup (age ≥ 65y) were not reported)

**Supplemental figure 1: PRISMA flow-diagram**

Records identified from:

Databases (k = 308)

Reference Screening (k = 4)

Trial Registers (k = 0)

Reports of trials included in previous review (k = 18)

Identification of new trials via other methods

Identification of new trials via databases and registers

Previous research

## Included

## Eligibility

## Screening

Records excluded, with reasons (k = 31)

Reports excluded

(k = 278)

Total trials included in review (k = 16)

Reports of total included trials (k = 21)

Full-text articles sought for eligibility (k = 18+34=52)

Records sought for retrieval (k = 34)

Records screened
(k = 312)

## Identification

**Supplemental figure 2: forest-plot DPP4 compared to placebo/no-treatment, discontinuation due to adverse events**
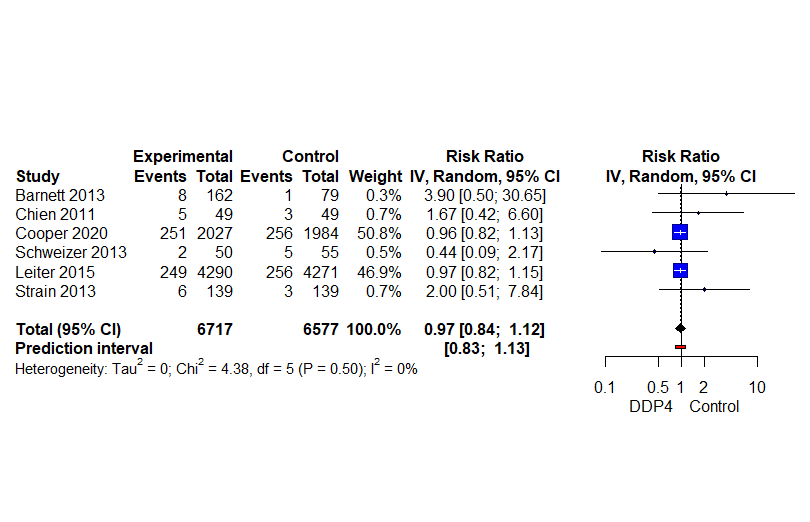


**Supplemental figure 3: forest-plot DPP4 compared to placebo/no-treatment, hospitalization**

**
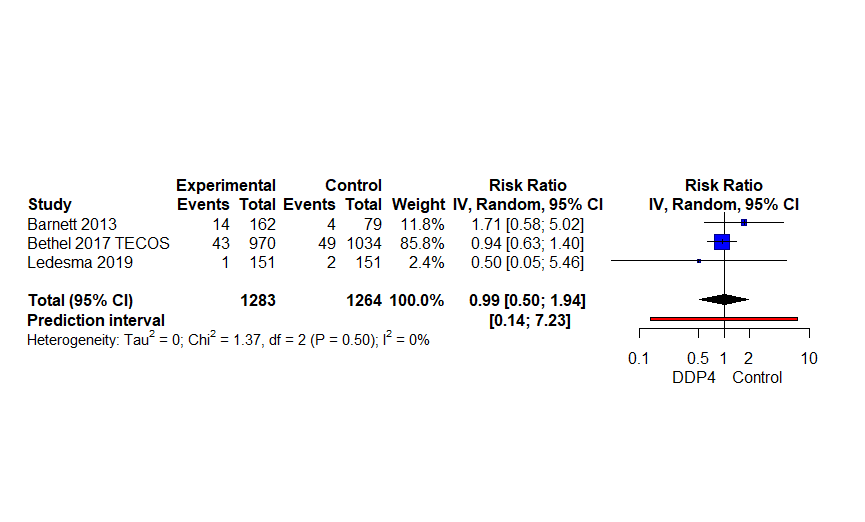
**

**Supplemental figure 4: forest-plot DPP4 compared to placebo/no-treatment, falls**


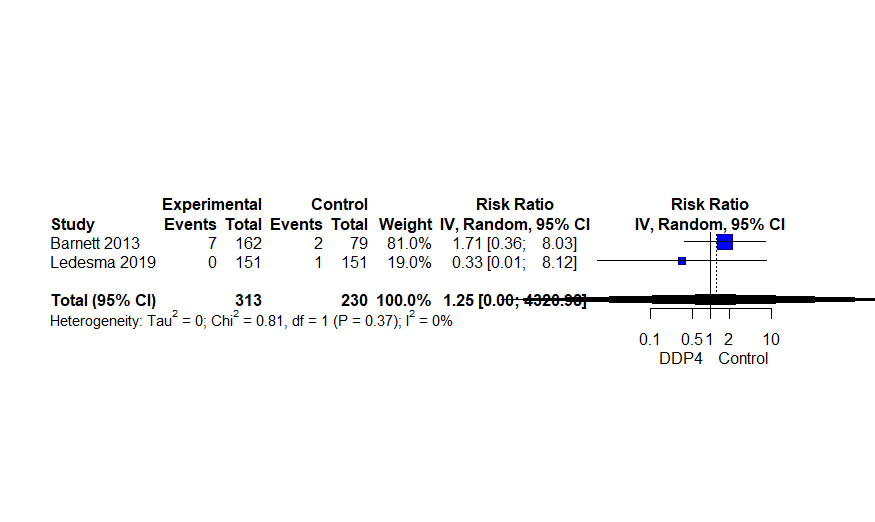


**Supplemental figure 5: forest-plot DPP4 compared to placebo/no-treatment, fractures**


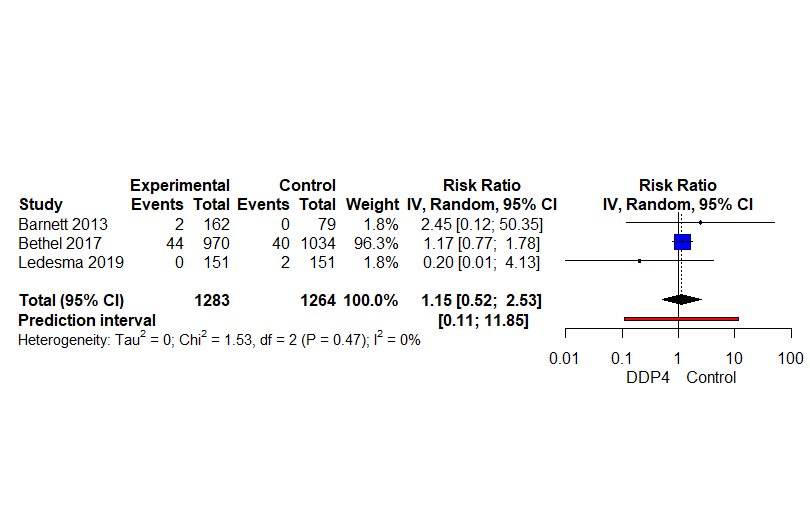


**Supplemental figure 6: forest-plot DPP4 compared to placebo/no-treatment, pancreatitis**


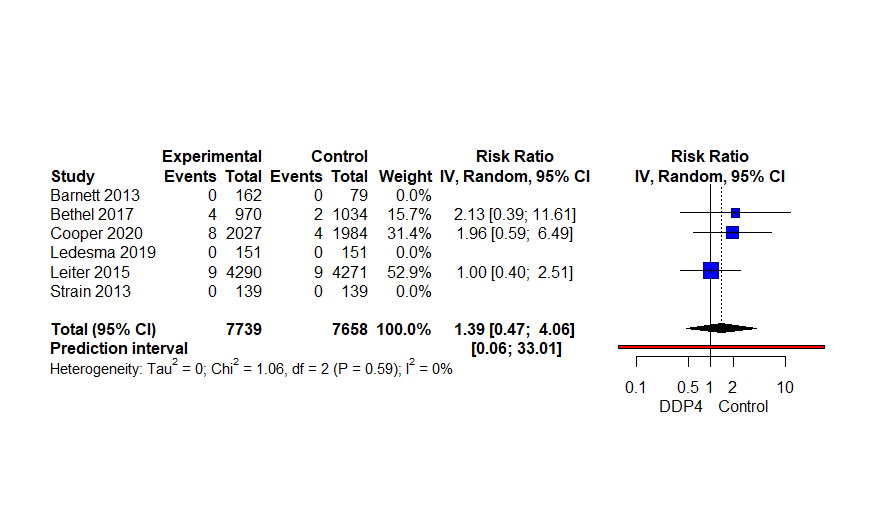


**Supplemental figure 7: forest-plot DPP4 compared to placebo/no-treatment, renal impairment**


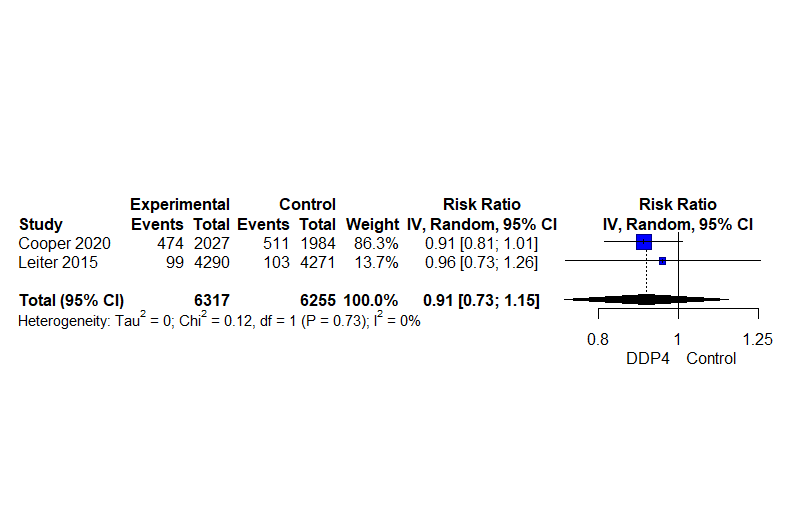


**Supplemental figure 8: forest-plot DPP4s compared to sulfonylureas, discontinuation due to adverse events**


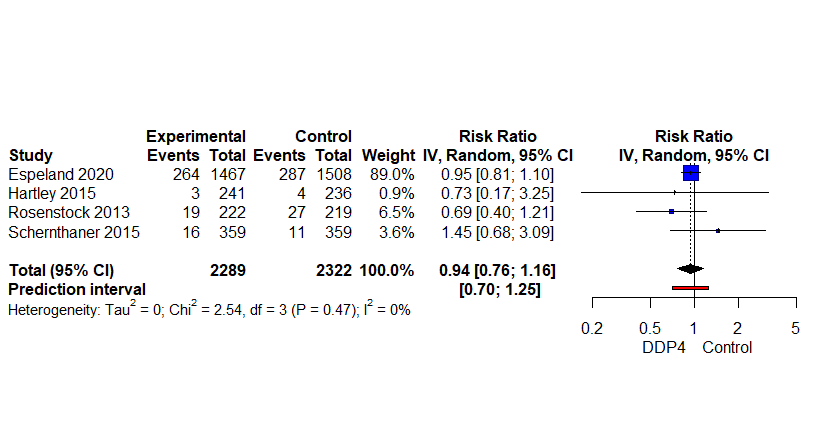


**Supplemental figure 9: forest-plot DPP4s compared to sulfonylureas, falls**


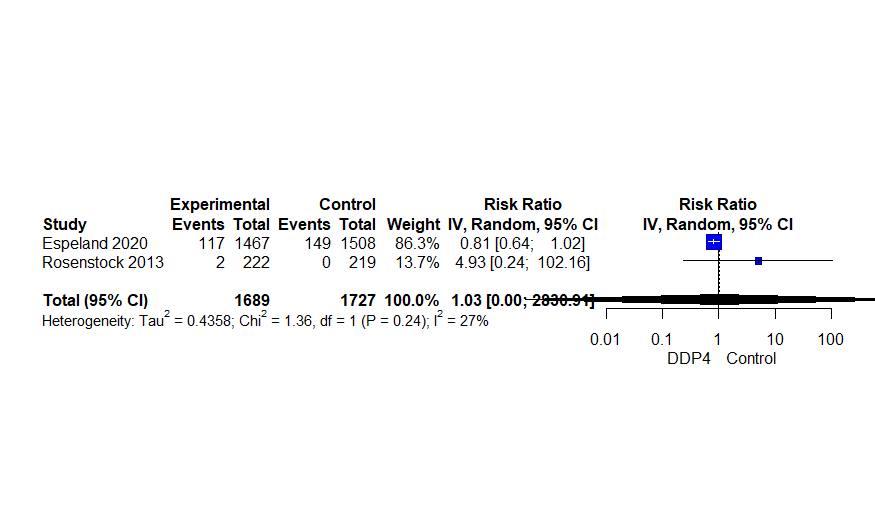


**Supplemental figure 10: forest-plot DPP4s compared to sulfonylureas, fractures**


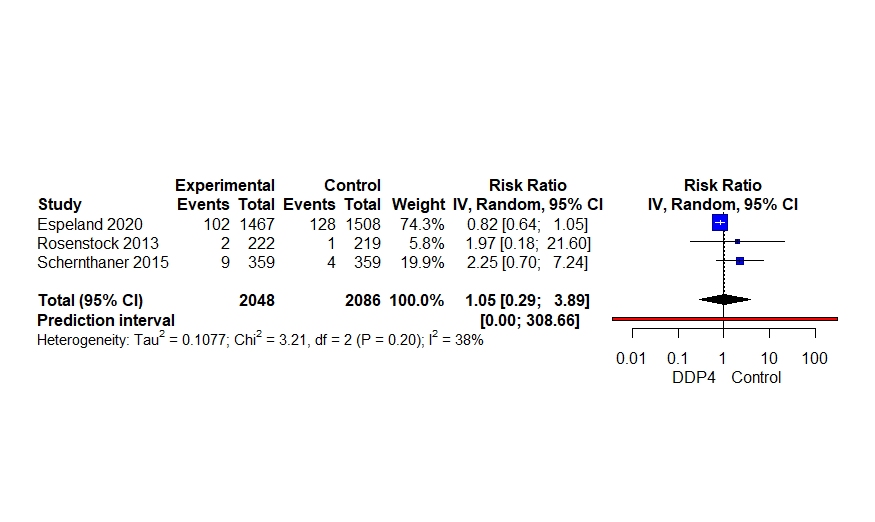


**Supplemental figure 11: forest-plot DPP4s compared to sulfonylureas, pancreatitis**


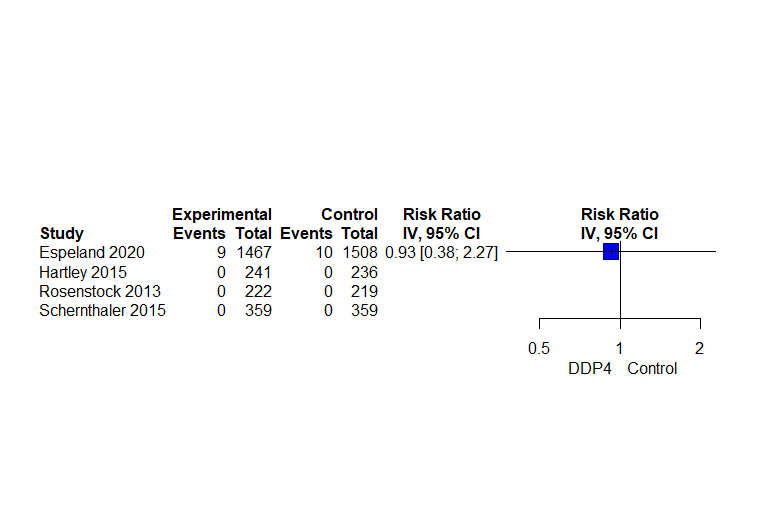


**Supplement V: DPP4s compared to sulfonylureas, pancreatitis (only beta-binomial model because all but one double zero studies)**

| **Events**  **experimental** | **Total experimental** | **Events control** | **Total**  **control** | **RR (95%CI)** |
| --- | --- | --- | --- | --- |
| 9 | 2289 | 10 | 2322 | 0.968 (0.045 to 20.85) |

**Supplement VI: results for outcomes for which only one study was available**

| ***DPP4 compared to sulphonylurea (n=2975)*** |
| --- |
| Hospitalization: RR 0.946 (95%CI 0.870 to 1.028) |
| ***DPP4 compared to sulphonylurea (n=441)*** |
| Renal impairment: only one event in the control group |

**Supplement VII: results for comparisons for which only one study was available**

| ***DPP4 compared to placebo (n=206)*** |
| --- |
| Mortality: no events  Any adverse event: RR 0.87 (95%CI 0.66 to 1.15)  Discontinuation due to adverse events: RR 1.70 (95%CI 0.42 to 6.93)  Hypoglycemia: no events  Fractures: to events in the placebo group  Renal impairment: one event in the placebo group |
| ***DPP4 compared to metformin (n=332)*** |
| Mortality: one event in the DPP4 group  Any adverse event: RR 0.88 (95%CI 0.70 to 1.11)  Discontinuation due to adverse events: RR 0.53 (95%CI 0.22 to 1.30)  Hypoglycemia: two events in the metformin group |
